# Near infra-red luminescent osmium labelled gold nanoparticles for cellular imaging and singlet oxygen generation†

**DOI:** 10.1039/d4nr01901f

**Published:** 2024-08-07

**Authors:** Luke S. Watson, Joseph Hughes, Salma T. Rafik, Asier R. Muguruza, Patricia M. Girio, Sarah O. Akponasa, Garret Rochford, Alexander J. MacRobert, Nikolas J. Hodges, Elnaz Yaghini, Zoe Pikramenou

**Affiliations:** a School of Chemistry, University of Birmingham Edgbaston B15 2TT UK z.pikramenou@bham.ac.uk; b Dept. of Surgical Biotechnology, Faculty of Medical Sciences, University College London London UK; c Department of Clinical Pharmacology, Faculty of Medicine, Alexandria University Alexandria 21516 Egypt; d School of Biosciences, University of Birmingham Edgbaston B15 2TT UK; e Doctoral Training Centre in Physical Sciences for Health, University of Birmingham Edgbaston B15 2TT UK

## Abstract

Osmium(ii) complexes have attractive properties for potential theranostic agents given their anticancer activitiy, their redox potentials favourable for biological transformations within cancer cells and their luminescence in the near infrared (NIR) region. To achieve localised detection and delivery, gold nanoparticles (AuNP) provide an attractive scaffold to attach multiple luminescent agents on a single particle and provide a multimodal platform for detection and loaclaised delivery. We have developed 13 nm and 25 nm AuNP decorated with an osmium complex based on 1,10-phenantholine and surface active bipyridine ligands, OsPhenSS for live cell imaging and singlet oxygen generation, notated as OsPhenSS·AuNP13 and OsPhenSS·AuNP25. The AuNP designs not only allow versatile modalities for localisation of the probe but also water solubility for the osmium metal complex. The osmium decorated nanoparticles OsPhenSS·AuNP13 and OsPhenSS·AuNP25 display characteristic NIR luminescence from the osmium(ii) ^3^MLCT at 785 nm in aqueous solutions with visible excitation. Upon incubation of the nanoparticles in lung cancer and breast carcinoma the luminescence signature of osmium and the gold reflectance reveal localisation in the cytoplasmic and perinuclear compartments. Excitation of the nanoparticles at 552 nm in the presence of a ROS indicator revealed a marked increase in the green fluorescence from the indicator, consistent with photo-induced ROS generation. The detection of singlet oxygen by time-resolved luminescence studies of the osmium and the nanoparticle probes further demonstrates the dual activity of the osmium-based nanoprobes for imaging and therapy. The introduction of gold nanoparticles for carrying osmium imaging probes allows a novel versatile strategy combining detection and localised therapies at the nanoscale.

## Abstract 2

Dual modality nanoprobes with enhanced theranostic properties are designed by decoration of gold nanoparticles with osmium complexes for imaging in the near-infrared and singlet oxygen production, leading to new localised therapy approaches.

## Graphical abstract

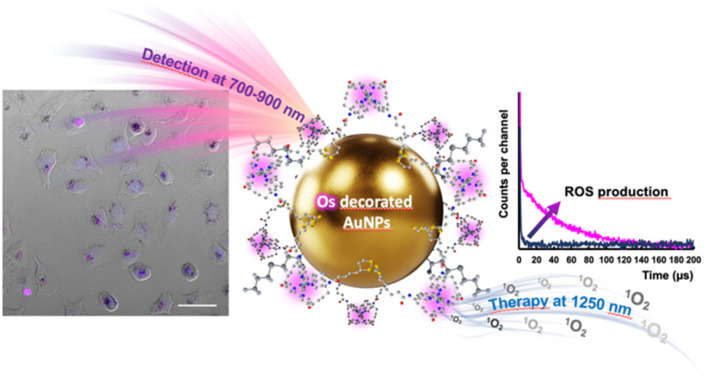

## 1. Introduction

Transition metal polypyridine complexes are attractive luminescence imaging probes for detection based on their photophysical properties arising from the Metal to Ligand Charge Transfer (MLCT) states and their versatility in the ligand functionalisation schemes for adaptation of their biological function.^1^ Whilst most popular probes rely on rhenium, ruthenium and iridium for cellular imaging, osmium metal complexes offer distinct advantages with detection in the near infra-red (NIR) of the spectrum, away from any interferences and ideal for the biological tissue window.^2,3^ Few osmium(ii) polypyridine complexes have been reported in cellular imaging applications,^4–6^ and recently pyridyl triazole complexes have been reported as probes with direct excitation of the ^3^MLCT energy band.^7^ Dinuclear Os(ii) complexes have been reported as a super-resolution NIR Simulated Emission Depletion (STED) probe for nuclear DNA.^8^ Transition metal complexes are also efficient photosensitisers^9–11^ due to their appreciable intensity in the ultraviolet and visible range of the spectrum and their long-lived ^3^MLCT state, which allows susceptibility towards quenching by oxygen in aerated solutions.^12,13^ The generation of singlet oxygen (^1^O_2_) which is an extremely reactive oxygen species (ROS) is a critical process in photodynamic therapy.^14^ The anticancer activity of osmium organometallic and coordination complexes has been well studied with many approaches^15,16^ adopting *in cellulo*^17^ photo-activation^18–20^ for localised therapeutic activity. It is well known that osmium compounds can exhibit higher oxidation states which may play a key role in biological redox potentials within cancerous cells.^21,22^ Efficient penetration of the photosensitiser probe into cancer cells is crucial to the effectiveness of the ROS to generate cell death, therefore intracellular tracking and imaging of the probe is important in order to evaluate the overall localisation and function. In this study we wish to introduce the employment of gold nanoparticles (AuNP) as a scaffold to deliver Os(ii) complexes inside cells, providing water solubility and overcoming cellular uptake of osmium luminescent probes for detection as well as ROS generation.

AuNP offer a versatile platform for the conjugation of lumophores, proteins and antibodies, and a plethora of advantages for imaging applications such as tuneable size, ease of synthesis, chemical inertness and biocompatibility.^23–25^ The high electron density of AuNP allows multimodal detection by Transmission Electron Microscopy (TEM) and light scattering in reflectance-based microscopy to provide imaging capabilities additional to conjugated lumophores. Organic photosensitisers have been conjugated to AuNP^26^ for production of ROS but to our knowledge, no osmium complex decorated AuNP has been utilised for its dual capabilities of live cell imaging and ROS generation in cancer cells. There have been couple reports of polypyridine Os(ii) complexes on nanoparticles either inside polymers or adsorbed onto 20 nm silver nanoparticles.^27,28^

**Fig. 1:**
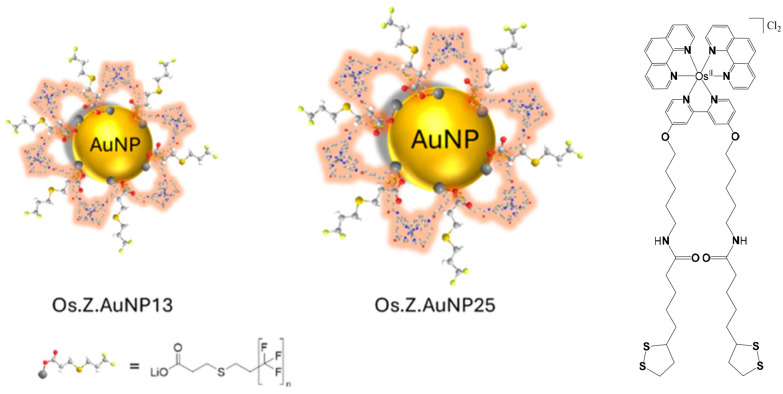
Schematic representation of nanoparticles decorated with OsPhenSS complex: OsPhenSS·AuNP13 and OsPhenSS·AuNP25.

The luminescence properties of probes near the AuNP surface can be strongly perturbed by plasmonic interactions that result in luminescence quenching at short distances from the surface and luminescence enhancement at longer distances.^29^ The attachment of luminescent metal probes to AuNP has been previously investigated for Ru(ii),^30,31^ Ir(iii)^32,33^ and Eu(iii)^34,35^ metal complexes. In this paper, we report AuNP of different sizes for optimising delivery of a Os(ii) complex as theranostic agent in cells for potential synergy of AuNP in plasmon-enhanced ^1^O_2_ production. The latter has been demonstrated as the production of ^1^O_2_ correlates with maximized scattering yield.^36^ The Os(ii) complex is designed with a long linker to optimise the position of the luminescent complex from the AuNP surface as previously shown (Fig. 1).^37–39^ The decoration of AuNP with osmium(ii) complexes is expected to increase the luminescence of the nanoprobe for imaging in cells in the far-red region of the spectrum. The internalisation of the osmium-decorated AuNP in A549 cancer cells has been examined and the ROS production has been evaluated.

## 2. Experimental

### 2.1 Materials

All solvents and chemicals used in synthesis were purchased from Fischer Scientific, VWR Chemicals, Sigma Aldrich and Alfa Aesar without further purification and deuterated NMR solvents were purchased from Goss Scientific. For gold nanoparticle (AuNP) synthesis, hydrogen tetrachloroaurate(iii) (Alfa-Aesar, UK, cat. no. 36400), sodium citrate tribasic dihydrate (Sigma-Aldrich, UK, cat. no. C8532), citric acid (Sigma-Aldrich, UK, cat. no. 251275), ethylenediaminetetraacetic acid (EDTA) (Sigma-Aldrich, UK, cat. no 03620), D_2_O (Sigma-Aldrich, UK, cat. no. 102494088). Dulbecco's modified Eagles medium (DMEM) (Sigma-Aldrich, UK, cat. no. D6046). PBS (Sigma-Aldrich, UK, cat. no. P2272). Hydromount mounting medium (National Diagnostics, UK, cat. no. HS-106). Paraformaldehyde solution, 4% in PBS (Thermo Scientific cat. no. 15670799). For ICP-MS: Nitric Acid, Trace Select, Honeywell cat. no. 02650-250 mL. Hydrochloric acid, Trace Select, Fisher cat. no. A508-P500. Water, Trace Select, Fluka cat. no. 95305-1L. DCF-DA (2′,7′-dichlorodihydrofluorescein diacetate, cat no. D6883) and Rose Bengal (cat no. 330000) were purchased from Sigma Aldrich, UK for ROS detection experiments.

### 2.2 Synthesis and characterisation of OsPhenSS

#### 2.2.a Synthesis of 4,4′-di(5-lipoamido-1-pentoxy)-2,2′-bipyridine

A solution of α-lipoic acid (0.72 g, 3.49 mmol) and 1-hydroxybenzotriazole hydrate (0.52 g, 3.88 mmol) in DMF (7 mL) was cooled to 0–5 °C, upon which 1-ethyl-3-(3-(dimethylamino)propyl) carbodiimide (EDC) (0.61 g, 0.7 mL, 3.96 mmol) was added and stirred, maintaining this temperature. The solution was allowed to warm to room temperature and stirred for a further hour. A solution of *N*-ethylmorpholine (0.29 g, 0.32 mL) and 4,4′-di-(5-amino-1-pentoxy)-2,2′-bipyridine (0.52 g, 1.45 mmol) in DMF (13 mL) was added to the reaction mixture and stirred overnight. The resulting cream precipitate was filtered and dried in the air and washed with DMF (2 × 10 mL) yielding the final product (0.80 g, 76%). *δ*H (400 MHz; CDCl_3_) 1.35–1.65 (12H, m, H8, 9, 15), 1.61–1.78 (8H, m, H14, 16), 1.79–1.96 (6H, m, H10, 18′), 2.17 (4H, t, 7.4, H13), 2.38–2.50 (2H, dq, 6.2, 12.4, H18), 3.02–3.14 (4H, m, H19), 3.29 (4H, q, 6.4, H11), 3.50–3.60 (2H, p, 6.7, H17), 4.13 (4H, t, 6.3, H7), 5.47 (2H, br s, NH), (6.82, 2H, dd, 2.5, 5.7, H5), 7.94 (2H, d, 2.5, H3) and 8.45 (2H, d, 5.7, H6). *δ*C (400 MHz; CDCl_3_) 23.40 (C9), 25.4 (C14), 28.6 (C15), 28.9 (C8), 29.4 (C10), 34.6 (C16), 36.5 (C13), 38.5 (C19), 39.3 (C11), 40.2 (C18), 56.4 (C17), 67.7 (C7), 106.8 (C3), 111.3 (C5), 150.2 (C6), 157.8 (C2), 166.1 (C4) and 172.7 (C12). MS [ESI^+^] *m*/*z* 735.3 (M + H).

#### 2.2.b Synthesis of bis-(1,10-phenantholine)-osmium dichloride

Os(phen)_2_Cl_2_ was synthesised with modification according to Chao *et al.*^40^ An ethylene glycol solution of OsCl_3_·0.5H_2_O (210 mg, 0.57 mmol) and 1, 10-phenanthroline (210 mg, 1.16 mmol) were brought to reflux under N_2_ atmosphere for 1 h. After cooling to room temperature, the solution was added to an aqueous solution containing Na_2_S_2_O_4_ (4.55 g, 25 mL) and kept in a refrigerator overnight. The dark precipitate was collected by vacuum filtration, washed with water and diethyl ether and directly used in the following reaction. MS [ESI^+^] *m*/*z* 622.0 [M]^+^.

#### 2.2.c Synthesis of OsPhenSS

Os(phen)_2_Cl_2_ (39.5 mg, 0.0635 mmol) and 4,4′-di(5-lipoamido-1-pentoxy)-2,2′-bipyridine (46.23 mg, 0.0629 mmol) was suspended in ethylene glycol (20 mL) and heated to 120 °C overnight. The black/brown solution was cooled to room temperature and 20 mL of deionised water was added. Saturated NH_4_PF_6_ in water (1 mL) was added and stirred for 20 min at RT and filtrated to give a black precipitate. The precipitate was washed with copious amounts of ice-cold water and followed by washes of diethyl ether (3 × 20 mL). The solid was dissolved in a minimal amount of acetonitrile and the solvent was removed *in vacuo* to give a black powder. An alumina column in MeCN was performed and then reduced *in vacuo* to yield the desired complex, OsPhenSS as the PF_6_ salt (23 mg, 72%). MS [ESI^+^] *m*/*z* 643.2 [M − 2PF_6_]^2+^. *λ*_max_ (MeCN)/nm (*ε*/dm^3^ mol^−1^ cm^−1^) 225 (61 000), 266 (63 400), 343sh (9200), and 485 (17 600) *δ*H (400 MHz; CD_3_CN) 1.48 (20H, m, H8, 9, 14, 15, 16), 1.80 (6H, m, H10, 18′), 2.07 (4H, td, 2.5, 7.2, H13), 2.37 (2H, dddd, 1.3, 5.6, 6.9, 13.3, H18), 3.08 (8H, m, H11, 19), 3.53 (2H, ddd, 2.3, 4.4, 8.5, H17), 4.18 (4H, td, 1.9, 6.5, H7), 6.39 (2H, s, NH), 6.74 (2H, dd, 2.6, 6.6, H5), 7.26 (2H, dd, 1.0, 6.5, H6), 7.43 (2H, dd, 5.4, 8.2, Hb′), 7.74 (2H, dd, 5.4, 8.2, Hb), 7.77 (2H, dd, 1.2, 5.4, Ha′), 8.04 (2H, d, 2.5, H3), 8.22 (6H, m, Hf/f′/a), 8.27 (2H, dd, 1.2, 8.2, Hc), 8.37 (2H, dd, 1.2, 8.2, Hc′). *δ*C (400 MHz; CD_3_CN) 23.6 (C9), 26.3 (C15), 28.9 (C8), 29.5 (C14), 29.9 (C10), 35.3 (C16), 36.7 (C13), 39.2 (C19), 39.3 (C11), 41.1 (C18), 57.5 (C17), 70.7 (C7), 112.4 (C3), 115.3 (C5), 126.9 (Cb′), 127.1 (Cb), 129.2 (Cf/f′), 132.2 (C4), 136.9 (Cc/c′), 151.4 (Cd/d′), 152.8 (C6), 153.0 (Ca′), 153.2 (Ca), 161.0 (C2), 167.2 (Ce/e′) and 173.5 (C12). NMR assignments were confirmed by COSY, HSQC and HMBC.

#### 2.2.d Photophysical measurements

UV-Vis spectra were collected by a Varian Cary 60 or 5000 spectrometers. Steady-state and timeresolved luminescence studies were performed by an Edinburgh Instruments FLS920 spectrometer equipped with 450 W Xe lamp for steady-state and an EPL 445 nm pulse diode laser, analysed by FAST software. Detection was performed with a liquid nitrogen cooled Hamamatsu PMT R5509-72. The luminescence quantum efficiency of OsPhenSS was calculated to be 1.3% with reference to indocyanine green (ICG) in aerated DMSO at RT:^41^
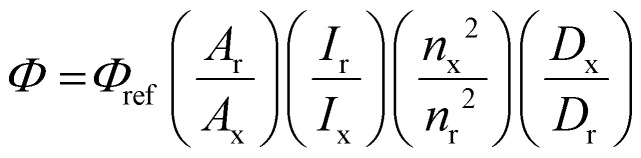
where *Φ* is the quantum yield, *A* is the absorbance, *I* is the intensity of the lamp, *n* is the refractive index of the solvent, *D* is the integral of the peak, x is the sample and r is the reference.

#### 2.2.e Detection of singlet oxygen NIR phosphorescence

The singlet oxygen phosphorescence at 1270 nm was detected using time-resolved photon counting from aerated solutions in quartz cuvettes. For detection in the near-IR, a thermoelectrically cooled photomultiplier (model H10330-45, Hamamatsu Photonics Ltd, Hertfordshire, UK) was used, and the emission was collected *via* a series of lenses from the cuvette in combination with a long-pass and a band-pass filter centred at 1270 nm (Interferenzoptik Electronik GmbH, Germany). Excitation was carried out using a pulsed 532 nm Nd: YAG laser (3 kHz repetition rate and 3 ns pulse length, Lumanova GmbH, Hannover, Germany) with the beam axis aligned orthogonally to the collection optics, and a fast photodiode (Becker-Hickl, Berlin, Germany) was used to synchronize the laser pulse with the photon counting system. Calibrated neutral density filters were used to attenuate the laser power. The photon counting detection equipment consisted of a multiscaler board (MSA-300, Becker-Hickl, Berlin, Germany) and a pre-amplifier (Becker-Hickl, Berlin, Germany). Integrated time-resolved phosphorescence traces were analysed using FluoFit software (PicoQuant GmbH, Berlin, Germany) to extract the singlet oxygen decay lifetime and amplitudes. OsPhenSS was dissolved in deuterated methanol (CH_3_OD, Sigma-Aldrich, Gillingham, UK) and solutions were placed in 1 cm quartz cuvettes. For measurement of singlet oxygen emission from the NPs, solutions were prepared in D_2_O. Since H_2_O rapidly quenches singlet oxygen, it was critical to minimise the H_2_O content when preparing the solution in D_2_O, as described in section 2.3.e. To minimise attenuation of the 532 nm laser excitation beam by the SPR band of the Au NPs, a short pathlength quartz cuvette (2 mm) was employed. The addition of sodium azide (Sigma-Aldrich, Gillingham, UK) dissolved in D_2_O served as a positive control since azide is an efficient singlet oxygen quencher.

#### 2.2.f Singlet oxygen quantum yield

Determination of the singlet oxygen quantum yield (*Φ*_Δ_) of OsPhenSS was carried out in deuterated methanol using Rose Bengal (*Φ*_Δ_ = 0.79)^42^ as the standard reference compound with optically matched solution absorbances (<0.1) at the laser wavelength of 532 nm. For matching the absorbance *versus* the reference compound, spectra were measured using a UV/Vis spectrophotometer (V-630, Jasco (UK) Ltd) with 1 cm quartz cuvettes. The relative value of *Φ*_Δ_ for OsPhenSS was calculated *vs.* Rose Bengal using standard zero-point intercept analysis of the singlet oxygen signal as a function of incident laser power.

### 2.3 Synthesis and characterisation of gold nanoparticles

#### 2.3.a Citrate-capped AuNP13

The synthesis was based on a previously published method^43^ which was slightly modified. A solution of trisodium citrate (60.3 mg, 0.21 mmol), citric acid (13.6 mg, 0.07 mmol) and ethylenediaminetetraacetic acid (EDTA) (1.6 mg, 0.004 mmol) in deionised water (100 mL) was stirred vigorously and brought to reflux. A preheated solution (80 °C) of hydrogen tetrachloroaurate (HAuCl_4_) (8.5 mg, 0.022 mmol) in deionised water (25 mL) was rapidly added to the centre of the vortex. After a 30-minute reflux, the heat was turned off and the solution was allowed to slowly cool to room temperature to form a 1.6 nM solution. *λ*_max_ (H_2_O) 517 nm (SPR). Diameter/nm: 14 ± 3 (DLS number distribution), PDI = 0.06, ζ-potential/mV = −35 ± 4.

#### 2.3.b Citrate-capped AuNP13 in D_2_O

The protocol for synthesizing AuNP13 was based on the published method by Turkevich *et al.*^43^ The glassware used was washed with aqua regia (HCl : HNO_3_, 3 : 1) and dried in the oven before use. A solution of trisodium citrate dihydrate (30.3 mg, 0.11 mmol), citric acid (6.8 mg, 0.035 mmol) and ethylenediaminetetraacetic acid (EDTA) (0.8 mg, 0.002 mmol) in 50 mL D_2_O was vigorously stirred and brought to reflux under a nitrogen atmosphere. This was left to reflux for 15 min, before the rapid addition of a solution of gold(iii)chloride trihydrate (HAuCl_4_._3_H_2_O) (4.3 mg, 0.010 mmol) in 12.5 mL of D_2_O. The solution was heated at reflux for a further 15 min, the heat was then turned off and the solution was left to cool to room temperature, forming 1.6 nM AuNP13. *λ*_max_(D_2_O): 517 nm (SPR). Diameter 13 ± 3 nm (DLS number distribution), PDI = 0.07, ζ-potential/mV = −33 ± 8. The final solution was concentrated by centrifugation at 10 000*g* for 30 min. The supernatant was decanted, and the pellet was redispersed in D_2_O to form a 4.5 nM solution of AuNP13.

#### 2.3.c Citrate-capped AuNP25

The protocol was modified using a previously published method by Ziegler *et al.*^44^ Three stock solutions were prepared: a solution of 5 mM HAuCl_4_·3H_2_O; 57 mM ascorbic acid and 34 mM trisodium citrate dihydrate in MILLI-Q®. AuNP13 (36 mL, 2 nM) were diluted to 40 mL with MILLI-Q® water and vigorously stirred. The solutions used in the addition were diluted to 1 mM, 3 mM and 0.75 mM in MILLI-Q® water to 40 mL for HAuCl_4_·3H_2_O, ascorbic acid and trisodium citrate dihydrate respectively. The two solutions (HAuCl_4_·3H_2_O and ascorbic acid/trisodium citrate dihydrate) were simultaneously added dropwise over 10 min. The resultant solution was heated to reflux for 30 min forming a solution of 0.7 nM AuNP25. The reaction vessel was allowed to cool to RT and stored in the dark. UV Vis (H_2_O) *λ*_max_/nm = 519 (SPR). Diameter/nm = 21 ± 5 (DLS number distribution), PDI = 0.07, ζ-potential/mV = −34 ± 4.

#### 2.3.d OsPhenSS·AuNP13

A suspension of AuNP13 (2 mL, 2 nM) was centrifuged at 13 000*g* for 25 min, the supernatant was decanted, and the pellet was re-suspended in filtered MILLI-Q® water (1 mL) to form 4 nM AuNP13. Zonyl FSA solution (2.5% w/v) in deionised water (10 μL, 0.05 M) was added to 4 nM AuNP13 (1 mL), stirred for 20 min and centrifuged at 13 000*g* for 20 min. The supernatant was discarded, and the pellet was re-suspended in filtered MILLI-Q® water (350 μL) and combined to form Z·AuNP13 (1 mL, 9 nM). *λ*_max_ (H_2_O)/nm 518 (SPR). Diameter/nm = 20 ± 5 (DLS number distribution), PDI = 0.12, ζ-potential/mV = −49 ± 5. OsPhenSS (70 μL, 1 mM) was titrated into a 9 nM solution of Z·AuNP13 13 in 5 μL aliquots with continuous stirring. The nanoparticles OsPhenSS·AuNP13 were isolated by size exclusion chromatography. *λ*_max_ (H_2_O)/nm 521 (SPR). Diameter/nm = 18 ± 5 (DLS number distribution), PDI = 0.27, ζ-potential/mV = −39 ± 8.

#### 2.3.e OsPhenSS·AuNP13 in D_2_O

10% v/v Zonyl FSA solution in D_2_O (10 μL) was added to 4.5 nM AuNP13 (1 mL) and sonicated for 10 min. This was centrifuged at 10 000*g* for 30 min, the supernatant was decanted and the pellet was resuspended in D_2_O (1 mL) to form Z·AuNP13 in D_2_O. *λ*_max_(H_2_O): 519 nm (SPR). Diameter 21 ± 6 nm (DLS number distribution), PDI = 0.271. ζ-potential/mV = −76 ± 12 mV. OsPhenSS (56 μL, 1 mM) was titrated into a 4.5 nM (2 mL) solution of Z·AuNP13 and sonicated for 2 min between additions. The OsPhenSS·AuNP13 in D_2_O were isolated by size exclusion chromatography. *λ*_max_(H_2_O): 521 nm (SPR), Diameter 22 ± 6 (DLS number distribution), PDI = 0.265 ζ-potential/mV = −36 ± 8 mV.

#### 2.3.f OsPhenSS·AuNP25

A suspension of AuNP25 (2 mL, 0.7 nM) was centrifuged at 13 000*g* for 25 min, the supernatant was decanted, and the pellet was re-suspended in filtered MILLI-Q® water (1 mL) to form 1.4 nM AuNP25. Zonyl FSA solution (2.5% w/v) in deionised water (10 μL, 0.05 M) was added to 1.4 nM AuNP25 (1 mL), stirred for 20 min and centrifuged at 13 000*g* for 20 min. The supernatant was discarded, and the pellet was resuspended in filtered MILLI-Q® water (250 μL) and combined to form Z·AuNP25 (1 mL, 5 nM). *λ*_max_ (H_2_O)/nm 520 (SPR). Diameter/nm = 29 ± 8, PDI = 0.26, ζ-potential/mV = −42 ± 4 DLS sizing measurements were recorded with AuNP at pH 7, 1 nM in MILLI-Q® H_2_O. OsPhenSS (75 μL, 1 mM) was titrated into a 5 nM solution of Z·AuNP-25 in 5 μL aliquots with continuous stirring. Sephadex G-25 size exclusion chromatography was performed for the isolation of OsPhenSS·AuNP25. *λ*_max_ (H_2_O)/nm 524 (SPR). Diameter/nm = 27 ± 8 (DLS number distribution), PDI = 0.32, ζ-potential/mV = −36 ± 3.

### 2.4 Transmission electron microscopy (TEM) and dynamic light scattering (DLS)

TEM analysis was carried out for citrate-capped AuNP, OsPhen·AuNP13 and OsPhen·AuNP25 from 4 nM, 9 nM and 5 nM stock solutions respectively. For all metal-coated nanoparticle samples, a 1 in 1 dilution was performed in Milli-Q H_2_O yielding 4.5 nM and 2.5 nM respectively. From this, a 1 in-5 dilution was carried out on all nanoparticle samples in Milli-Q H_2_O and a 20 μL aliquot was dropped onto a 200 mesh formvar coated TEM grid and left to dry in the dark overnight. Imaging took place on a JEOL1400 transmission electron microscope (Electron Microscopy service, University of Birmingham). Dynamic Light Scattering (DLS) measurements were carried out on a Malvern Zetasizer nano ZSP in 1 mL quartz cuvettes with a 1 : 1 dilution factor. Measurements were recorded at pH 7 in MILLI-Q® H_2_O.

### 2.5 Inductively coupled mass spectrometry

Nanoparticle samples were analysed either an ICP-MS Agilent LC-ICP-MS (7500cx) at University of Warwick or a PerkinElmer 300X Nexion at University of Birmingham. To prepare the samples, the coated samples were diluted to 4.5 nM. A 20 μL aliquot was added to 80 μL Milli-Q H_2_O yielding a concentration of ∼1 nM. Samples were digested with ultra-pure aqua regia (300 μL). The samples were then diluted to 5 mL to reduce the aqua regia content to <4% with a 4% HNO_3_ solution containing ascorbic acid (50 mM), EDTA (50 mM) and thiourea (50 mM) to stop the production of the volatile osmium tetraoxide species. A series of gold and osmium standards were used for calibration.

### 2.6 Cell culture

The human lung cancer A549 was obtained from the European Collection of Authenticated Cell Cultures (ECACC catalogue number 86012804) cell line was maintained in Dulbecco's modified Eagle's medium (DMEM) supplemented with 10% w/v fetal bovine serum (FBS), l-glutamine (2 mM) and 1% penicillin (100 units per mL) streptomycin (100 μg per mL) hereafter called complete media. Cells were cultured in 20 mL of complete media in vented T75 flasks at 37 °C in a humidified 95% air: 5% CO_2_ atmosphere. Cells were routinely sub-cultured from high confluency using a standard trypLE protocol. The human breast carcinoma cell line (MCF-7) was obtained from ECACC, (catalogue number 86012803). Cells were cultured in Dulbecco's modified Eagle's medium/Nutrient Mixture F12 Ham (Sigma Aldrich, Dorset, UK), supplemented with 10% w/v Foetal bovine serum (Gibco, UK) and 1% penicillin (100 units per mL) and streptomycin (100 μg mL^−1^) (Gibco, UK). Dosing of A549 cells was performed in 35 mm dishes with a 10 mm glass insert. Cells were seeded at 100 000 cells per dish in 1 mL complete media. Before dosing with AuNP cells were washed in warm PBS and cell media was replaced with 900 μL fresh media. Samples of OsPhenSS·AuNP13 (100 μL, 9 nM) and OsPhenSS·AuNP25 (100 μL, 5 nM) were diluted upon addition to cell media to final concentrations as indicated in the specific experiments. For confocal microscopy of cells, cell media was removed and cells were washed twice in warm PBS. For Hoechst nuclear staining 20 μM of a 20 mM stock solution of Hoechst 3328 in PBS was added for 10 min in the dark. The cells were washed and phenol red-free cell media was added (1 mL per well). To provide additional validation of cellular uptake and for carrying out ROS generation imaging, we used the human breast carcinoma MCF-7 cell line, which was obtained from the European Collection of Authenticated Cell Cultures (ECACC). Cells were cultured in Dulbecco's modified Eagle's medium/Nutrient Mixture F12 Ham (Sigma Aldrich, Dorset, UK), supplemented with 10% fetal bovine serum (Gibco, UK) and 1% penicillin (5000 units per mL) and streptomycin (5000 μg mL^−1^) (Gibco, UK).

### 2.7 Cell viability assay *via* MTT

Cells were seeded at a density of 5000 cells per well in a 96-well plate. Nanoparticle concentrations were varied across the plate by serial dilution factor in six technical replicates containing a positive (1% v/v Triton) and negative control (cell media). Cells were dosed for 24 h with varying concentrations of OsPhen·AuNP (9 nM–0.14 nM) and washed with phenol red-free media. The cells were then further incubated with (3-(4,5-dimethylthiazol-2-yl)-2,5-diphenyltetrazolium bromide) (MTT, final concentration 0.5 mg per mL) reagent in Dulbecco's Modified Eagle Medium (DMEM) for 4 h and then removed completely. DMSO was added to each well to dissolve the purple precipitate and absorption at 590 ± 30 nm using a plate reader (Tecan Infinite 200).

### 2.8 Confocal microscopy

Confocal microscopy of fixed A549 cells was performed using a Leica TCS SP8 upright confocal laser scanning system using a 64× oil immersion objective lens. 405 nm, 458 nm, 488 nm, 633 nm solid-state laser lines were used. Confocal microscopy of live A549 cells was performed using the same system with a 40× ceramic H_2_O immersion dip objective lens. Cells were fixed by the addition of 4% paraformaldehyde (PFA) to each well (1 mL per well) for 15 min in the dark. PFA was removed and cells were washed twice in PBS (0.1 M, pH 7.4). Coverslips were removed and mounted on a droplet of hydromount media (National Diagnostics, UK) on glass slides, and stored flat in the dark for at least 24 h at 4 °C before imaging. Live cells were imaged immediately after the washing. Fluorescence imaging of live MCF-7 cells was carried out using an upright Leica SP8 confocal microscope. Cells were plated into 35 mm diameter Petri dishes equipped with a glass coverslip base (Fluorodish, WPI, Herts, UK) 24 h before the addition of the nanoparticles. The nanoparticles were incubated with the cells for up to 24 h, following by washing and addition of phenol-red free medium added, and imaging was carried out using an immersion objective with excitation at 552 nm and detection in the red/NIR from 680–770 nm.

For fluorescence of imaging photo-induced generation of reactive oxygen species (ROS), MCF-7 cells were incubated with the ROS probe 2′,7-dichlorodihydrofluorescein (DCF-DA, Sigma Aldrich, Dorset, UK). DCF-DA at 10 μM was added to the incubation medium 2 h before imaging. Confocal luminescence imaging was carried out using excitation at 488 nm and emission detection of green fluorescence from 510–550 nm. For on-stage photoexcitation to induce intracellular ROS generation, illumination was carried out at 552 nm for 300 s with an incident power <1 mW to selectively excite the osmium(ii) complex but not the DCF-DA probe dye, thereby minimising autooxidation of DCF-DA. Cells were re-imaged within 5 min of the on-stage illumination.

## 3. Results and discussion

**Fig. 2:**
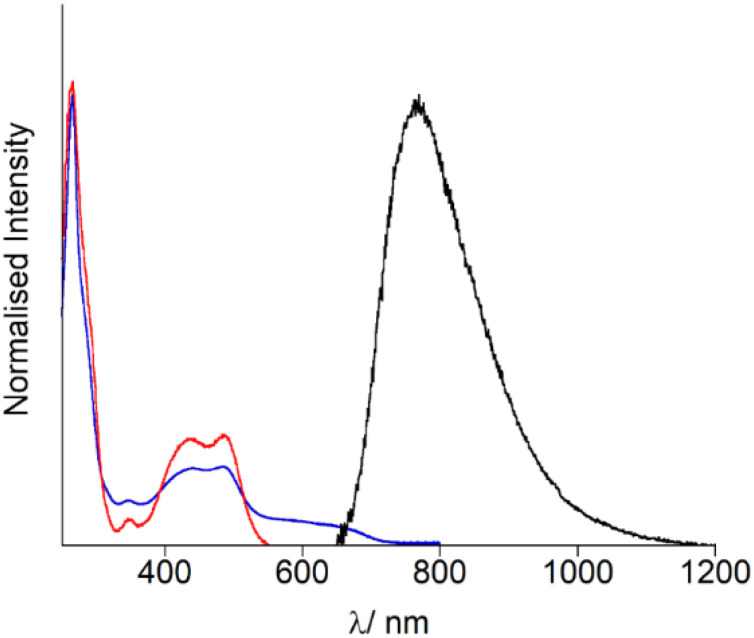
Optical spectroscopy of OsPhenSS in aerated aqueous solution (2% CH_3_CN) UV-Vis absorption spectrum: blue, luminescence excitation spectrum (*λ*_em_ = 770 nm) red, and luminescence emission spectrum (*λ*_exc_ = 488 nm); black.

Firstly, the photophysical properties of OsPhenSS in solution were evaluated for further comparisons with the decorated AuNP. UV-Vis spectroscopy studies (Fig. 2, Table 1) show an intense ligand-centred (LC) ^1^(π–π*) band at 266 nm arising from the phenantholine character, a weak intraligand charge transfer (ILCT) band at 340 nm with ^1^MLCT (d–π*) band arising between 400–550 nm with an estimated *λ*_max_ at 485 nm (Fig. 2, Table 1). This is in accordance with other osmium bis-phenantholine derivatives.^45–48^ Additionally, the spin forbidden absorption for ^3^MLCT is also observed at 550–750 nm due to Os(ii) high spin orbit coupling.^49–51^OsPhenSS displays luminescence from the ^3^MLCT centred at 770 nm in aerated water. The broad emission is significantly red-shifted by 62 nm in comparison to [Os(phen)_3_]^2+^ suggesting that the bulky ligand moieties may have a stabilising effect on the excited state which is in agreement with other similar osmium(ii) complexes.^52,53^ The NIR emission profile of OsPhenSS exceeds other bis-phenantholine osmium complexes and majority of bis-bipyridine and bis-terpyridine osmium complexes,^5–7,20,40,54–61^ although a few with emission profiles exceeding 800 nm have been reported.^19^ To examine the effect of fluorosurfactant Zonyl FSA, which stablises nanoparticle formation, to OsPhenSS independently, we studied the luminescence properties upon the addition of excess of Zonyl FSA. No shift of the luminescence *λ*_max_ was observed.

**Table 1:** Absorption and luminescence properties of OsPhenSS in aerated solution and Os(ii)-decorated AuNP

	Absorption *λ*_max_/nm (*ε*/M^−1^ cm^−1^)	Luminescence *λ*_max_/nm
OsPhenSS	225 (61 000), 266 (63 400), 343sh (9200), 485 (17 600)	770
OsPhenSS + Zonyl FSA	225 (59 000), 266 (62 000), 343sh (9200), 485 (17 000)	770
OsPhen·AuNP13	521	785
OsPhen·AuNP25	524	785

However, the luminescence lifetime of OsPhenSS was increased to 46 ns by the presence of Zonyl FSA in aerated water over the free complex in aerated water at 36 ns. This enhancement in lifetime is attributed to the interaction of Zonyl FSA with OsPhenSS resulting in protection of ^3^O_2_ quenching.^62–64^ Solvent interactions have been shown to lower the rate of diffusion of ^3^O_2_ resulting in stronger emission and luminescent lifetimes.^37,65^ The singlet oxygen quantum yield of OsPhenSS was calculated as 0.20 ± 0.01. The singlet oxygen decay lifetime was measured as 31 μs which is in good agreement with the literature.^66^

**Fig. 3:**
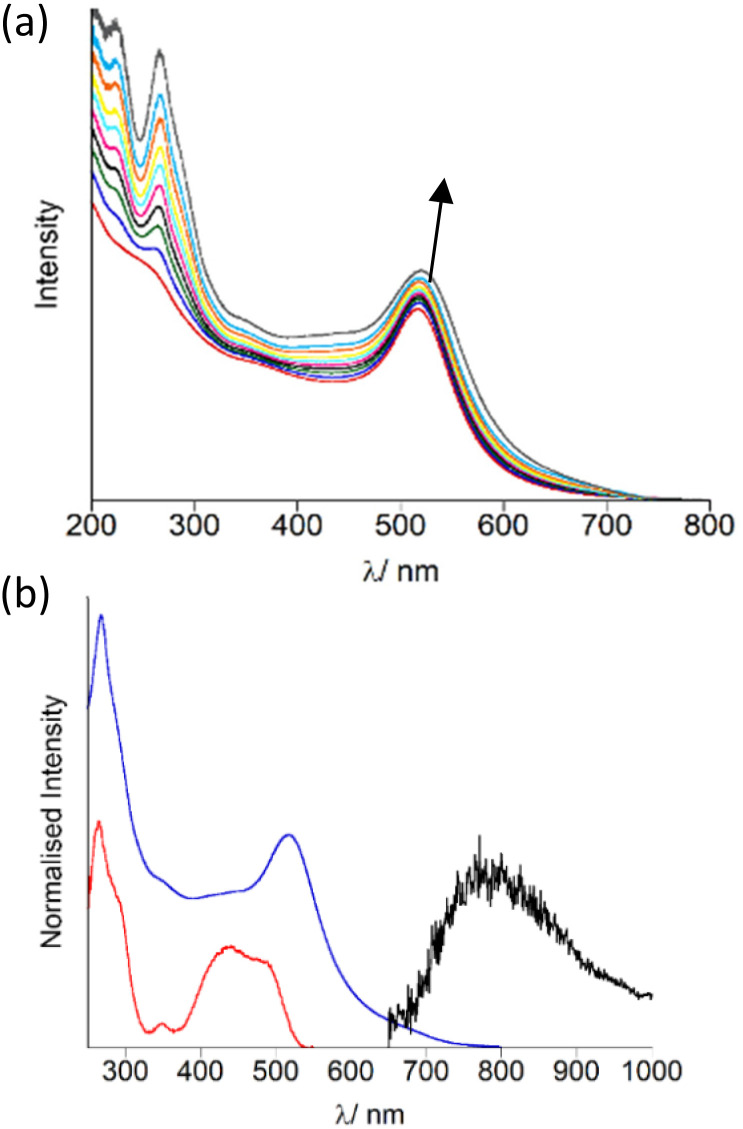
Monitoring OsPhenSS coating on AuNP13 and optical spectra of isolated OsPhen·AuNP13 in water. (a) Titration of 5 μL quantities of 1 mM OsPhenSS (5 μL aliquots of 1 mM) into 4.5 nM Z·AuNP13 (4.5 nM) in aerated water and (b) isolated OsPhen·AuNP13 UV-Vis absorption spectrum: blue, excitation spectrum (*λ*_em_ = 770 nm): red, and luminescence emission spectrum (*λ*_exc_ = 488 nm), black.

The AuNP were decorated with OsPhenSS by titration of 5 μL quantities (1 mM) of OsPhenSS to a suspension of 4.5 nM Z·AuNP13 and 1.5 nM Z·AuNP25 monitoring the shift of the surface plasmon resonance (SPR) band characteristic of the changes on the surface of the AuNP, in order to determine full saturation of the surface (Fig. 3a and Fig. S15† for AuNP25). Additions of 35 μL and 18 μL of 1 mM OsPhenSS into 4.5 nM Z·AuNP13 and 1.5 nM Z·AuNP25 resulted in shifts of 4 and 5 nm of the SPR respectively. The particles were purified through a G25 Sephadex column and the luminescence properties were evaluated (Fig. 3b, Table 1).

After isolation of the aqueous suspension of OsPhenSS·AuNP13 and OsPhenSS·AuNP25, the SPR band was not shifted indicating only excess complex was removed during the isolation process. This is confirmed though the reduction of the LC peak at 266 nm. The excitation spectra mirror the absorption profile of the free complex in aerated water showing the characteristic ^1^MLCT band at 488 nm (Fig. 3b). Excitation of ^1^MLCT at 488 nm of OsPhenSS·AuNP13 and OsPhenSS·AuNP25 leads to an emission signal at 785 nm. Upon excitation at 488 nm OsPhenSS·AuNP13 and OsPhenSS·AuNP25, a 15 nm red shift is observed in their broad ^3^MLCT emission profiles which can be attributed to the change of the environment surrounding the metal complex and the influence of the SPR (Fig. S18†).^52,67^ The OsPhenSS·AuNP13 and OsPhenSS·AuNP25 are spherical in nature with mean diameters of 14 ± 3 nm and 21 ± 5 nm as performed by DLS, respectively. TEM confirmed average sizes of 14 ± 1 nm (*n* = 50) for OsPhenSS·AuNP13 and 21 ± 1 nm (*n* = 50) for OsPhenSS·AuNP25 which is in good agreement with DLS data (Fig. S16†). Aqueous solutions of the OsPhenSS·AuNP13 display a luminescence lifetime of 55 ns, equating to a 19 ns increase in comparison to the free OsPhenSS complex in aerated water and a small increase of 9 ns in comparison with the influence of the presence of surfactant. This effect is consistent with similar polypyridine-coated ruthenium complexes attached to AuNP.^30–34,37,39,68,69^ Inductively Couple Mass spectrometry (ICP-MS) studies of the OsPhenSS·AuNP13 estimate ∼1800 complexes per single AuNP, and ∼4800 for OsPhenSS·AuNP25 demonstrating high loading on the nanoparticle surface ideal for cellular imaging.

**Fig. 4:**
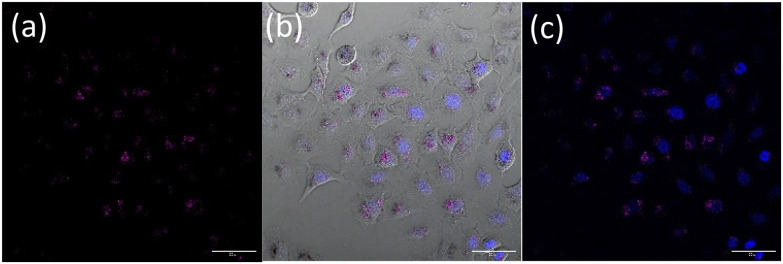
Representative live cell images of A549 cells treated with 0.9 nM OsPhenSS·AuNP13 for 18 h. (a) Osmium emission (purple); (b) brightfield overlapped with osmium emission (purple) and Hoescht 3368 (blue) and (c) overlap of osmium emission (purple) and Hoescht 3368 (blue). Channels: Osmium emission (*λ*_exc_ = 488 nm, *λ*_em_ = 650–800 nm); Hoescht 3368 *λ*_exc_ = 402 nm, *λ*_em_ = 420–470 nm. The scale bar is 50 μm.

Nanoparticle uptake and localization experiments were performed with an optimized nanoparticle dosage based on (Fig. S19†) in lung cancer cell line A549. Additionally MTT assays in a non-cancerous lung cell were also performed (Fig. S20†). Confocal microscopy of live A549 cells incubated with OsPhenSS·AuNP13 for 18 h revealed cytoplasmic and perinuclear localisation of the nanoparticles within the cells (Fig. 4). Localisation was confirmed using a nuclear stain Hoescht 3368.

**Fig. 5:**
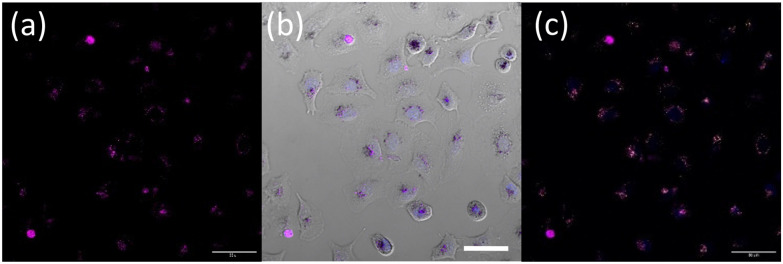
Representative live cell images of A549 cells treated with 0.2 nM OsPhenSS·AuNP25 for 18 h. (a) Purple channel, osmium emission, (b) brightfield overlapped with both osmium emission (purple) and Hoescht 3368 (blue) and (c) overlap of osmium emission (purple), Hoescht 3368 (blue) and reflectance (yellow). Channels: Osmium emission (*λ*_exc_ = 488 nm, *λ*_em_ = 650–800 nm); Hoescht 3368 *λ*_exc_ = 405 nm, *λ*_em_ 420–470 nm; reflectance (*λ*_exc_ = 633 nm, *λ*_em_ = 623–643 nm). The scale bar is 50 μm.

Furthermore, upon incubation of A549 cells with OsPhenSS·AuNP25 nanoparticles, osmium luminescence was observed within the cytoplasm and perinuclear area of live cells (Fig. 5, Fig. S21†), similar to OsPhenSS·AuNP13 nanoparticles. Reflectance microscopy is also informative of localisation due to the scattering signal from the dense AuNP which is more prominent in the case of OsPhenSS·AuNP25. Colocalization between the osmium signal and gold reflectance (Fig. 5 and Fig. S22†) confirmed that the recorded luminescence signal arises from the OsPhenSS complexes attached to AuNP25. Imaging studies of fixed cell imaging at 4 h showed localisation throughout the cells alongside perinuclear localisation rather than in a single compartment (Fig. S23†).

**Fig. 6:**
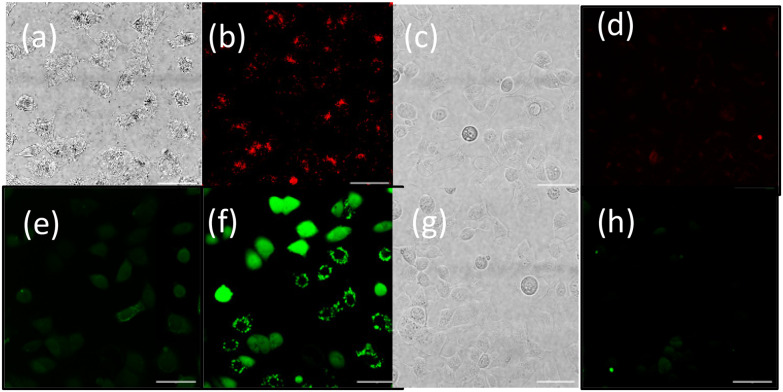
Representative live cell images of MCF-7 cells for luminescence (a)–(d) and ROS generation studies (e)–(h), incubated with OsPhenSS·AuNP25. (a) and (b) and OsPhenSS·AuNP25 with DCF-DA (e) and (f) (24 h, [OsPhenSS·AuNP25] = 0.1 nM) and controls (c) and (d) and (g) and (h). (a) Bright-field image of cells in (b); (b) osmium luminescence (red channel); (c) bright-field image of cells without nanoparticles; (d) red channel control without nanoparticles. (e) green channel fluorescence before on-stage illumination of 300 s at 552 nm, of same field as (f); (f) green channel after on-stage illumination of 300 s at 552 nm, using same intensity scale as 6 (e) and same field; (g) bright-field image of cells incubated with DCF-DA only for control shown in (h) and (h) green channel fluorescence image of cells incubated with DCF-DA only of same field as (g) after on-stage illumination of 300 s at 552 nm; Channels: Osmium red channel emission (*λ*_exc_ = 552 nm, *λ*_em_ = 680–780 nm); DCF-DA green channel (*λ*_exc_ = 488, *λ*_em_ = 510–550 nm); scale bar is 50 μm.

To provide further validation of cellular uptake and red/NIR luminescence detection of the OsPhenSS·AuNP25, confocal imaging was also carried out in the human breast carcinoma cell line MCF-7 (Fig. 6). Imaging analysis showed punctate intracellular luminescence in live MCF-7 cells (Fig. 6b) after 24 h incubation with OsPhenSS·AuNP25 (Fig. 6B and Fig. S24†). In serum-free medium. Incubation for 4 h in contrast gave a lower signal by a factor of three. Cells were also incubated with the intracellular ROS probe 2′,7′-dichlorodihydrofluorescein diacetate DCF-DA, which emits green fluorescence upon oxidation. On-stage illumination at 552 nm was used to selectively photoexcite the osmium complex. A comparison of the images (Fig. 6(e) and (f) for pre and post illumination) shows a marked increase in the green fluorescence, consistent with photo-induced intracellular ROS generation in the photosensitised cells. The initial punctate intracellular localisation is consistent with endocytic uptake of the nanoparticle which would be the expected uptake route.^30,32^ However intracellular ROS generation will not be confined to endolysosomes since the vesicle membranes can be ruptured by ROS such as singlet oxygen resulting in redistribution of the NP within the cytosol.^70^ Although we did not attempt to quantify superoxide generation we note that even small quantum yields of this species can result in oxidative damage *via* Type 1 photocalalytic interactions.^71,72^

Examination of bright-field images of cells photosensitised with OsPhenSS·AuNP25 showed that illumination at 402 nm induced morphological changes in the cells (*e.g.* swelling) consistent with cellular injury whereas illumination of cells without exposure to the nanoparticles elicited no evident changes. These observations are consistent with photooxidative damage *via* ROS generation. No morphological changes were observed in non-irradiated cells (Fig. S22 and S25†).

**Fig. 7:**
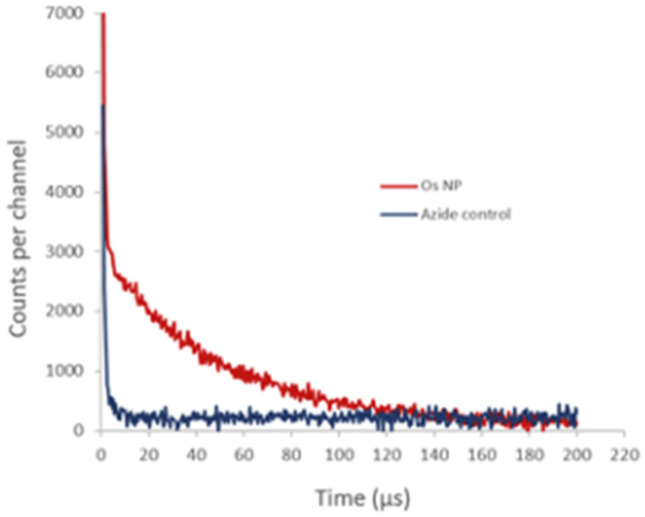
Time-resolved singlet oxygen NIR phosphorescence decays from OsPhenSS·AuNP13 in D_2_O 4.5 nM (*λ*_det_ = 1270 ± 20 nm). Red: without the addition of azide; blue: with the addition of azide (5 mM). The initial short-lived signal is due to the residual NIR tail of fluorescence from the osmium signal. In the presence of azide, which is a physical quencher of singlet oxygen, the NIR phosphorescence tail is suppressed.

We also studied the detection of singlet oxygen phosphorescence directly from OsPhenss·AuNP13 (Fig. 7). Since singlet oxygen is quenched efficiently by H_2_O giving a decay lifetime of only ∼3 μs, we prepared AuNP *in situ* in D_2_O to minimise any H_2_O content (as described in Materials and methods) and then performed the coating with OsPhenSS as illustrated in Fig. 3. In D_2_O, the singlet oxygen lifetime is 20 times longer than for H_2_O and it is therefore much easier to detect the singlet oxygen phosphorescence using a D_2_O solution. This experiment was considerably more challenging technically than the singlet oxygen studies using the OsPhenSS complex due to competing optical absorption from the AuNP at 532 nm and we had to use a short pathlength cuvette so that the laser excitation beam was not attenuated significantly. This short path length however restricted the signal that could be collected by the relay lens optics coupled to the detector. The use of a longer wavelength excitation beyond the SPR band would alleviate this problem.

Fig. 7 shows the NIR signal detected with and without the addition of azide which is an efficient quencher of singlet oxygen.^73^ In the absence of azide, we observed a decay with a lifetime of approximately 50 μs whereas, in the presence of azide, the longer-lived phosphorescence is quenched, which is consistent with a calculated lifetime of <1 μs at the 5 mM concentration of azide. The initial short-lived signal is unaffected by azide and is due to the residual NIR tail of photoluminescence from the OsPhenSS complex. This experiment was considerably more challenging technically than the singlet oxygen studies using the OsPhenSS complex itself due to competing optical absorption from the AuNP. Since the absorption due to the AuNP SPR is relatively high at the 532 nm laser excitation wavelength and dominates the lower OsPhenSS absorbance, we had to use a short pathlength cuvette so that the laser excitation beam was not attenuated significantly. This short path length however restricted the signal that could be collected by the relay lens optics coupled to the detector. The use of a longer wavelength excitation beyond the SPR band would alleviate this problem.

## 4. Conclusions

We have designed a luminescent osmium gold nanoparticle system for live cell imaging which shows NIR luminescence at 785 nm with low nanoparticle dosage in both lung cancer and human breast carcinoma cells while displaying localisation in perinuclear regions. Cells dosed with the osmium-decorated gold nanoparticles upon irradiation at 552 nm displayed a marked activity of ROS generation which shows the strong potential of osmium-coated gold nanoparticles with therapeutic activity. The detection of singlet oxygen phosphorescence was confirmed for the osmium complex and the coated gold nanoparticles, even though the absorbance of AuNP SPR and the low concentrations of osmium employed lead to weaker signal detection. To our knowledge, this is the first design of osmium-coated gold nanoparticles for live cell imaging and singlet oxygen generation which offer potential applications in oncotheranostics and non-oncological indications. The combination of the dye complex with the nanoparticle enables new routes in targeting and codelivery of other agents.

## Author contributions

The following authors contributed in the experimental parts as indicated: LW (synthesis, photophysical studies, imaging), JH (nanoparticles), ARM (cell imaging and analysis), PMG (nanoparticles preparation), SOA and GR (cell culture), STR (cell cultures and ROS generation) as well as in data analysis, project design and writing of the manuscript with support from NJH, AMR, EY and ZP.

## Data availability

The data supporting this article have been included as part of the ESI.†

## Conflicts of interest

There are no conflicts to declare.

## Supplementary Material

NR-016-D4NR01901F-s001

## References

[cit1] Lee L. C.-C., Lo K. K.-W. (2022). J. Am. Chem. Soc..

[cit2] Saeed H. K., Sreedharan S., Thomas J. A. (2020). Chem. Commun..

[cit3] Tan C.-P., Zhong Y.-M., Ji L.-N., Mao Z.-W. (2021). Chem. Sci..

[cit4] Byrne A., Dolan C., Moriarty R. D., Martin A., Neugebauer U., Forster R. J., Davies A., Volkov Y., Keyes T. E. (2015). Dalton Trans..

[cit5] Gkika K. S., Byrne A., Keyes T. E. (2019). Dalton Trans..

[cit6] Huang R., Feng F.-P., Huang C.-H., Mao L., Tang M., Yan Z.-Y., Shao B., Qin L., Xu T., Xue Y.-H., Zhu B.-Z. (2020). ACS Appl. Mater. Interfaces.

[cit7] Omar S. A. E., Scattergood P. A., McKenzie L. K., Jones C., Patmore N. J., Meijer A. J. H. M., Weinstein J. A., Rice C. R., Bryant H. E., Elliott P. I. P. (2018). Inorg. Chem..

[cit8] Chen Q., Jin C., Shao X., Guan R., Tian Z., Wang C., Liu F., Ling P., Guan J.-L., Ji L., Wang F., Chao H., Diao J. (2018). Small.

[cit9] Huang H., Banerjee S., Qiu K., Zhang P., Blacque O., Malcomson T., Paterson M. J., Clarkson G. J., Staniforth M., Stavros V. G., Gasser G., Chao H., Sadler P. J. (2019). Nat. Chem..

[cit10] Mulazzani Q. G., Ciano M., D'Angelantonio M., Venturi M., Rodgers M. A. J. (1988). J. Am. Chem. Soc..

[cit11] Monro S., Colón K. L., Yin H., Roque III J., Konda P., Gujar S., Thummel R. P., Lilge L., Cameron C. G., McFarland S. A. (2019). Chem. Rev..

[cit12] Brunschwig B., Sutin N. (1978). J. Am. Chem. Soc..

[cit13] Demas J. N., Harris E. W., McBride R. P. (1977). J. Am. Chem. Soc..

[cit14] Schweitzer C., Schmidt R. (2003). Chem. Rev..

[cit15] Hanif M., Babak M. V., Hartinger C. G. (2014). Drug Discovery Today.

[cit16] Pagliaricci N., Pettinari R., Marchetti F., Pettinari C., Cappellacci L., Tombesi A., Cuccioloni M., Hadiji M., Dyson P. J. (2022). Dalton Trans..

[cit17] Needham R. J., Sanchez-Cano C., Zhang X., Romero-Canelón I., Habtemariam A., Cooper M. S., Meszaros L., Clarkson G. J., Blower P. J., Sadler P. J. (2017). Angew. Chem., Int. Ed..

[cit18] Xue X., Fu Y., He L., Salassa L., He L.-F., Hao Y.-Y., Koh M. J., Soulié C., Needham R. J., Habtemariam A., Garino C., Lomachenko K. A., Su Z., Qian Y., Paterson M. J., Mao Z.-W., Liu H.-K., Sadler P. J. (2021). Inorg. Chem..

[cit19] Lazic S., Kaspler P., Shi G., Monro S., Sainuddin T., Forward S., Kasimova K., Hennigar R., Mandel A., McFarland S., Lilge L. (2017). Photochem. Photobiol..

[cit20] Ge C., Zhu J., Ouyang A., Lu N., Wang Y., Zhang Q., Zhang P. (2020). Inorg. Chem. Front..

[cit21] Berger G., Grauwet K., Zhang H., Hussey A. M., Nowicki M. O., Wang D. I., Chiocca E. A., Lawler S. E., Lippard S. J. (2018). Cancer Lett..

[cit22] Lu N., Deng Z., Gao J., Liang C., Xia H., Zhang P. (2022). Nat. Commun..

[cit23] Giljohann D. A., Seferos D. S., Daniel W. L., Massich M. D., Patel P. C., Mirkin C. A. (2010). Angew. Chem., Int. Ed. Engl..

[cit24] Murphy C. J., Gole A. M., Stone J. W., Sisco P. N., Alkilany A. M., Goldsmith E. C., Baxter S. C. (2008). Acc. Chem. Res..

[cit25] Kubota T., Kuroda S., Kanaya N., Morihiro T., Aoyama K., Kakiuchi Y., Kikuchi S., Nishizaki M., Kagawa S., Tazawa H., Fujiwara T. (2018). Nanomed. J..

[cit26] Mkhobongo B., Chandran R., Abrahamse H. (2022). Pharmaceutics.

[cit27] Yang T., Xia A., Liu Q., Shi M., Wu H., Xiong L., Huang C., Li F. (2011). J. Mater. Chem..

[cit28] Glomm W. R., Moses S. J., Brennaman M. K., Papanikolas J. M., Franzen S. (2005). J. Phys. Chem. B.

[cit29] Eustis S., El-Sayed M. A. (2006). Chem. Soc. Rev..

[cit30] Dosumu A. N., Claire S., Watson L. S., Girio P. M., Osborne S. A. M., Pikramenou Z., Hodges N. J. (2021). JACS Au.

[cit31] Rogers N. J., Claire S., Harris R. M., Farabi S., Zikeli G., Styles I. B., Hodges N. J., Pikramenou Z. (2014). Chem. Commun..

[cit32] King S. M., Claire S., Teixeira R. I., Dosumu A. N., Carrod A. J., Dehghani H., Hannon M. J., Ward A. D., Bicknell R., Botchway S. W., Hodges N. J., Pikramenou Z. (2018). J. Am. Chem. Soc..

[cit33] Rogers N. J., Jeffery H. C., Claire S., Lewis D. J., Zikeli G., Hodges N. J., Egginton S., Nash G. B., Pikramenou Z. (2017). Nanomedicine.

[cit34] Davies A., Lewis D. J., Watson S. P., Thomas S. G., Pikramenou Z. (2012). Proc. Natl. Acad. Sci. U. S. A..

[cit35] Lewis D. J., Pikramenou Z. (2014). Coord. Chem. Rev..

[cit36] Macia N., Kabanov V., Côté-Cyr M., Heyne B. (2019). J. Phys. Chem. Lett..

[cit37] Osborne S. A. M., Pikramenou Z. (2015). Faraday Discuss..

[cit38] Adams S. J., Lewis D. J., Preece J. A., Pikramenou Z. (2014). ACS Appl. Mater. Interfaces.

[cit39] Martinez-Calvo M., Orange K. N., Elmes R. B. P., la Cour Poulsen B., Williams D. C., Gunnlaugsson T. (2016). Nanoscale.

[cit40] Zhang P., Wang Y., Qiu K., Zhao Z., Hu R., He C., Zhang Q., Chao H. (2017). Chem. Commun..

[cit41] Kurahashi T., Iwatsuki K., Onishi T., Arai T., Teranishi K., Hirata H. (2016). J. Biomed. Opt..

[cit42] Tanielian C., Wolff C. (1995). J. Phys. Chem..

[cit43] Turkevich J., Stevenson P. C., Hillier J. (1951). Discuss. Faraday Soc..

[cit44] Ziegler C., Eychmüller A. (2011). J. Phys. Chem. C.

[cit45] Kober E. M., Caspar J. V., Sullivan B. P., Meyer T. J. (1988). Inorg. Chem..

[cit46] Pankuch B. J., Lacky D. E., Crosby G. A. (1980). J. Phys. Chem..

[cit47] Roque J. A., Barrett P. C., Cole H. D., Lifshits L. M., Shi G., Monro S., von Dohlen D., Kim S., Russo N., Deep G., Cameron C. G., Alberto M. E., McFarland S. A. (2020). Chem. Sci..

[cit48] Omar S. A. E., Scattergood P. A., McKenzie L. K., Bryant H. E., Weinstein J. A., Elliott P. I. P. (2016). Molecules.

[cit49] Zych D., Slodek A., Matuszczyk D., Golba S. (2018). Eur. J. Inorg. Chem..

[cit50] Ito A., Knight T. E., Stewart D. J., Brennaman M. K., Meyer T. J. (2014). J. Phys. Chem. A.

[cit51] Kober E. M., Meyer T. J. (1982). Inorg. Chem..

[cit52] Kober E. M., Caspar J. V., Lumpkin R. S., Meyer T. J. (1986). J. Phys. Chem..

[cit53] Lumpkin R. S., Kober E. M., Worl L. A., Murtaza Z., Meyer T. J. (1990). J. Phys. Chem..

[cit54] Dröge F., Noakes F. F., Archer S. A., Sreedharan S., Raza A., Robertson C. C., MacNeil S., Haycock J. W., Carson H., Meijer A. J. H. M., Smythe C. G. W., Bernardino de la Serna J., Dietzek-Ivanšić B., Thomas J. A. (2021). J. Am. Chem. Soc..

[cit55] Zhang P., Huang H. (2018). Dalton Trans..

[cit56] Mardanya S., Karmakar S., Mondal D., Baitalik S. (2016). Inorg. Chem..

[cit57] Hwang K.-C., Chen J.-L., Chi Y., Lin C.-W., Cheng Y.-M., Lee G.-H., Chou P.-T., Lin S.-Y., Shu C.-F. (2008). Inorg. Chem..

[cit58] Ge C., Huang H., Wang Y., Zhao H., Zhang P., Zhang Q. (2018). ACS Appl. Bio Mater..

[cit59] Chu W.-K., Yiu S.-M., Ko C.-C. (2014). Organometallics.

[cit60] Scattergood P. A., Sinopoli A., Elliott P. I. P. (2017). Coord. Chem. Rev..

[cit61] Carlson B., Phelan G. D., Kaminsky W., Dalton L., Jiang X., Liu S., Jen A. K. Y. (2002). J. Am. Chem. Soc..

[cit62] El Garah M., Marets N., Mauro M., Aliprandi A., Bonacchi S., De Cola L., Ciesielski A., Bulach V., Hosseini M. W., Samorì P. (2015). J. Am. Chem. Soc..

[cit63] Liu J.-L., Zhang J.-Q., Tang Z.-L., Zhuo Y., Chai Y.-Q., Yuan R. (2019). Chem. Sci..

[cit64] Zanarini S., Rampazzo E., Ciana L. D., Marcaccio M., Marzocchi E., Montalti M., Paolucci F., Prodi L. (2009). J. Am. Chem. Soc..

[cit65] Huang R., Huang C.-H., Shao J., Zhu B.-Z. (2019). J. Phys. Chem. Lett..

[cit66] Bregnhøj M., Westberg M., Jensen F., Ogilby P. R. (2016). Phys. Chem. Chem. Phys..

[cit67] Ansari S. A., Khan M. M., Ansari M. O., Cho M. H. (2015). New J. Chem..

[cit68] Caballero A. B., Cardo L., Claire S., Craig J. S., Hodges N. J., Vladyka A., Albrecht T., Rochford L. A., Pikramenou Z., Hannon M. J. (2019). Chem. Sci..

[cit69] Lamansky S., Djurovich P., Murphy D., Abdel-Razzaq F., Kwong R., Tsyba I., Bortz M., Mui B., Bau R., Thompson M. E. (2001). Inorg. Chem..

[cit70] Yaghini E., Giuntini F., Eggleston I. M., Suhling K., Seifalian A. M., MacRobert A. J. (2014). Small.

[cit71] Mroz P., Bhaumik J., Dogutan D. K., Aly Z., Kamal Z., Khalid L., Kee H. L., Bocian D. F., Holten D., Lindsey J. S., Hamblin M. R. (2009). Cancer Lett..

[cit72] Yaghini E., Pirker K. F., Kay C. W. M., Seifalian A. M., MacRobert A. J. (2014). Small.

[cit73] Li M. Y., Cline C. S., Koker E. B., Carmichael H. H., Chignell C. F., Bilski P. (2001). Photochem. Photobiol..

