# Supplementary material for: Near infra-red luminescent osmium labelled gold nanoparticles for cellular imaging and singlet oxygen generation
Source: Nanoscale. 2024 Aug 7;16(35):16500–9. doi: 10.1039/d4nr01901f (PMC11331564; doi:10.1039/d4nr01901f)
Supplement: NR-016-D4NR01901F-s001 [file NR-016-D4NR01901F-s001.pdf]

Supplementary Information (SI) for Nanoscale.
This journal is © The Royal Society of Chemistry 2024

Supporting information

Near Infra-Red Luminescent Osmium Labelled Gold Nanoparticles for Cellular Imaging and Singlet Oxygen Generation

Luke S. Watson, [a] Joseph Hughes,[a] Salma T. Rafik,[b, c] Asier R. Muguruza,[a] Patricia M. Girio,[a,e] Sarah O. Akponasa,[a, d] Garret Rochford,[d] Alexander J. MacRobert,[b] Nikolas J. Hodges,[d] Elnaz Yaghini[b] and Zoe Pikramenou[a]

[a.] *School of Chemistry,*[e] Doctoral Training Centre in Physical Sciences for Health, *University of Birmingham, Edgbaston, B15 2TT, United Kingdom.*
[b.] *Dept. of Surgical Biotechnology, Faculty of Medical Sciences, University College London, London, United Kingdom.*
[c.] *Department of Clinical Pharmacology, Faculty of Medicine, Alexandria University, Alexandria 21516, Egypt.*
[d.] *School of Biosciences, University of Birmingham, Edgbaston, B15 2TT, United Kingdom.*

*Corresponding author: z.pikramenou@bham.ac.uk

This file includes:

Table of Contents — Page number

BpySS Characterisation — S 2

$Os(phen)_2Cl_2$ Characterisation — S 4

OsPhenSS Characterisation — S 5

Photophysical Characterisation of OsPhenSS in presence of fluorosurfactant — S10

Data summary of gold nanoparticles' characterisation data — S11

Photophysical characterisation of OsPhenSS gold nanoparticles — S13

Cell viability assays of OsPhenSS gold nanoparticles in live A549 cells — S13

Additional confocal images of z-stacks of **OsPhenSS•AuNP25** — S15

Calculation of number (*N*) of gold atoms per nanoparticle — S17

BpySS Characterisation

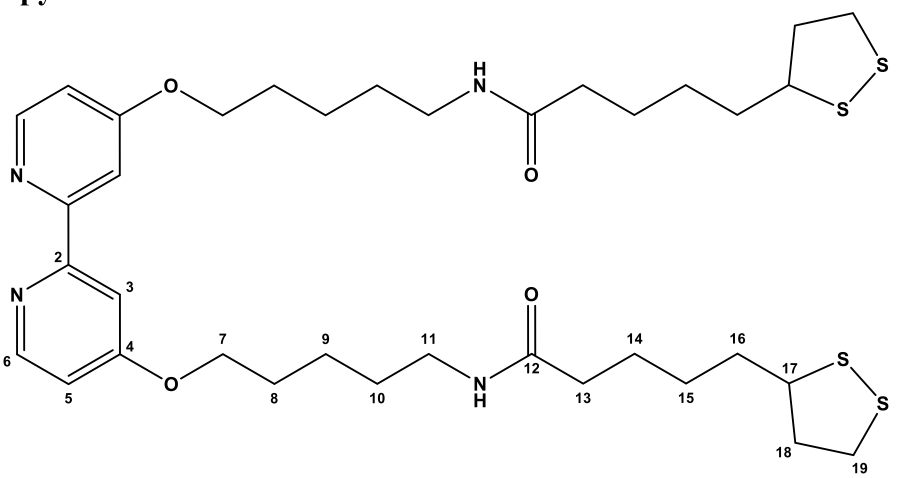

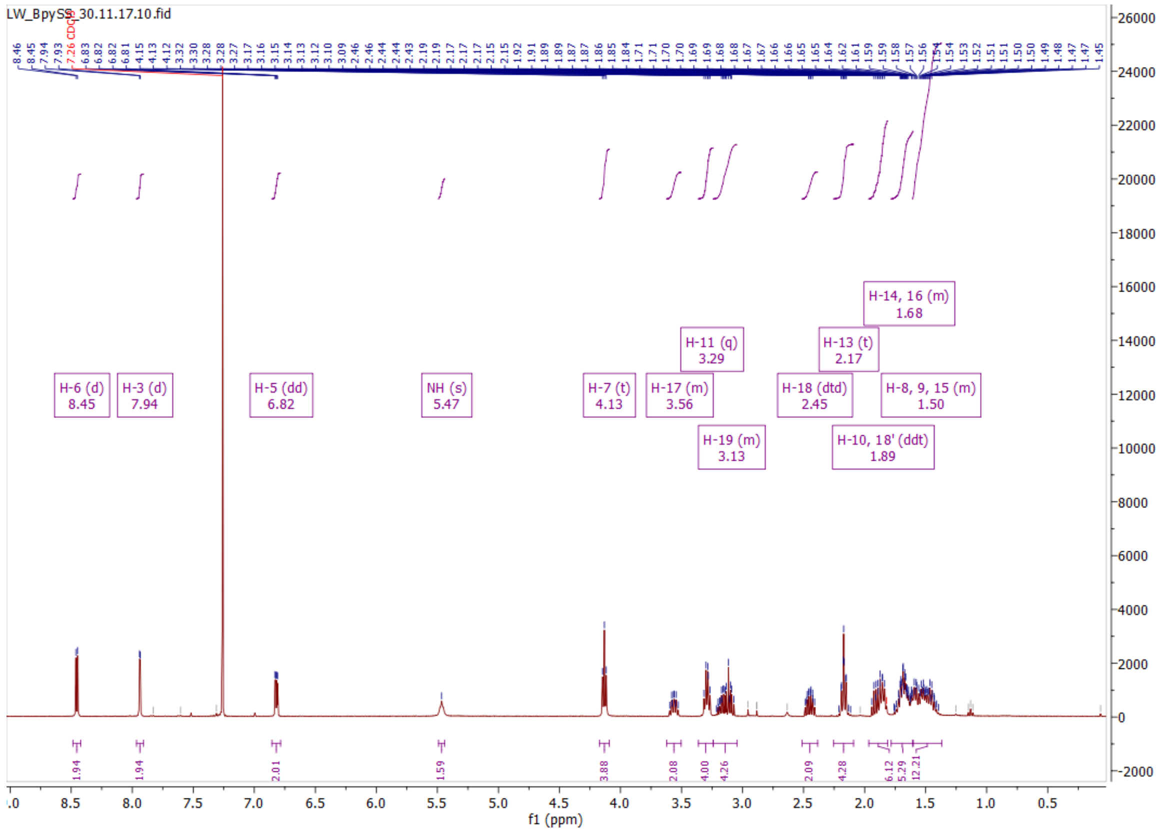

Figure S1. ^{1}H NMR spectrum of BpySS in $CDCl_3$.

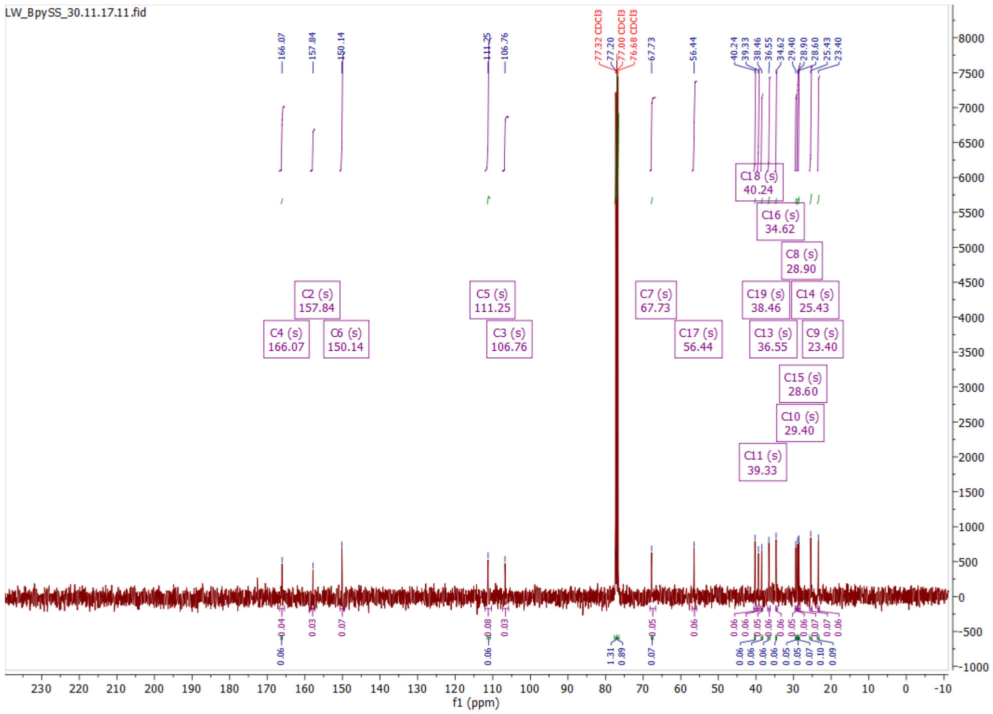

Figure S2. ^{13}C NMR spectrum of BpySS in $CDCl_3$.

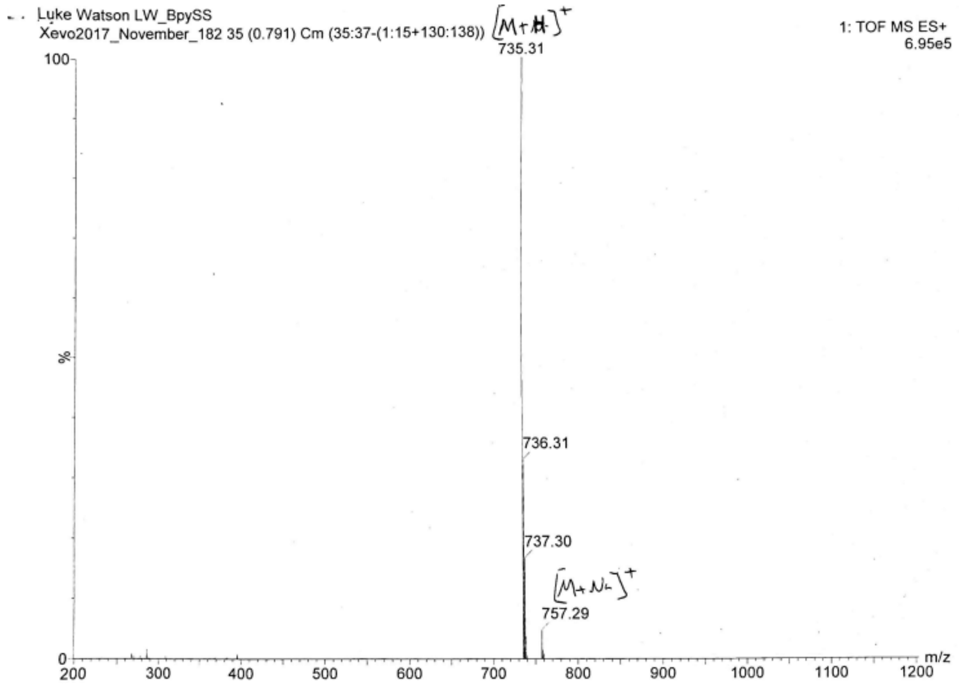

Figure S3. ESI (+) mass spectrum of BpySS.

Os(phen)$_2$Cl$_2$ Characterisation

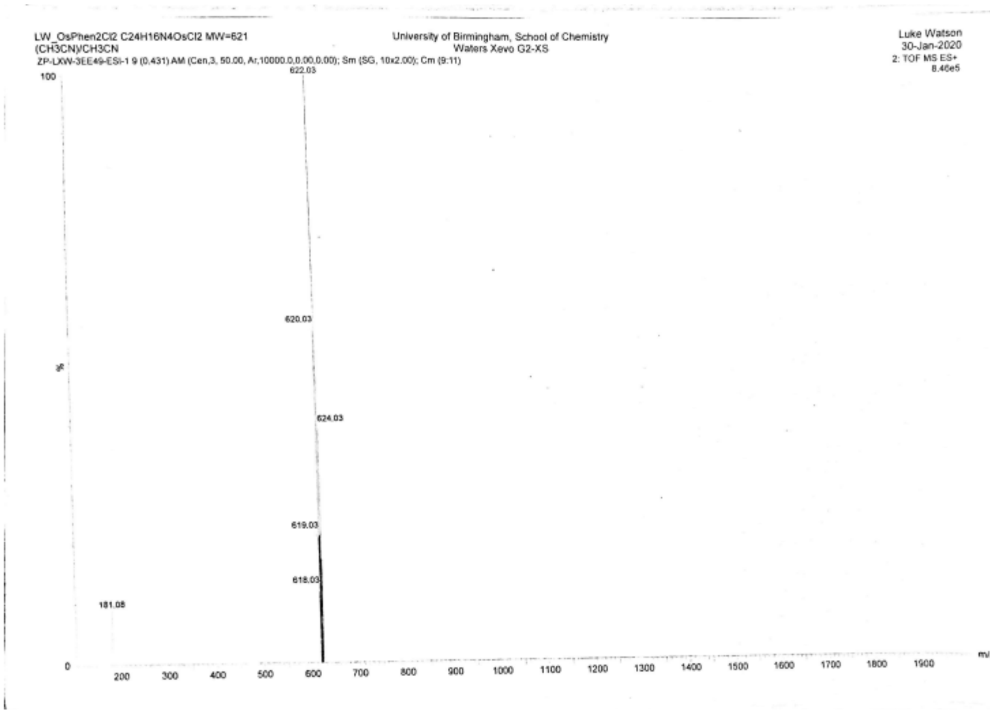

Figure S4. ESI(+) mass spectrum of Os(Phen)$_2$Cl$_2$.

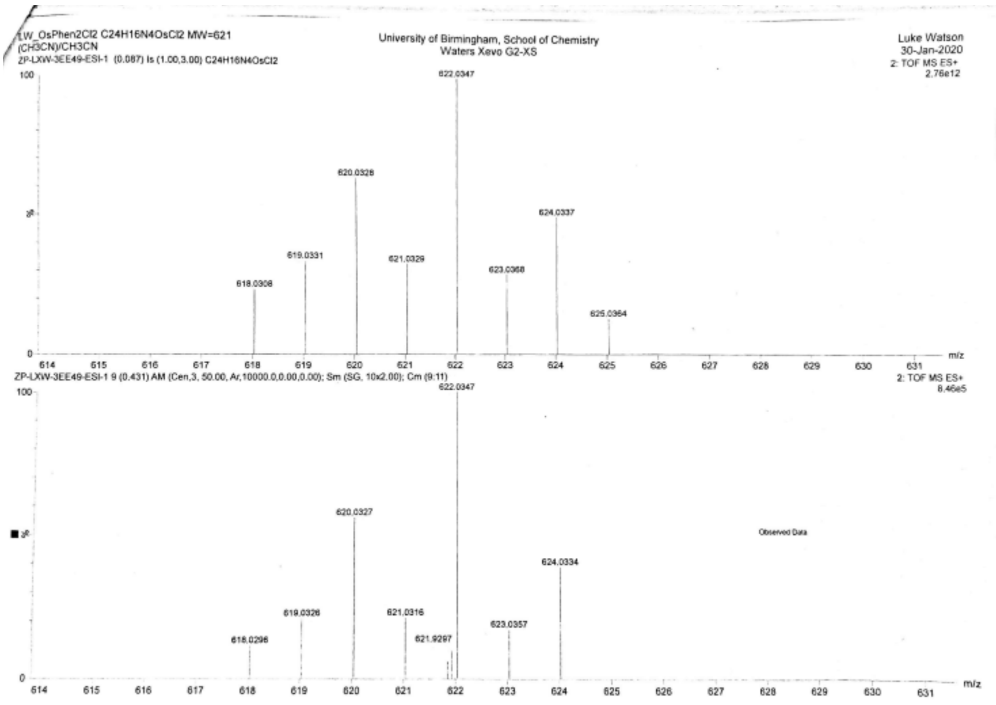

Figure S5. ESI (+) mass spectrum of Os(Phen)$_2$Cl$_2$ of experimental mass vs theoretical mass.

OsPhenSS Characterisation

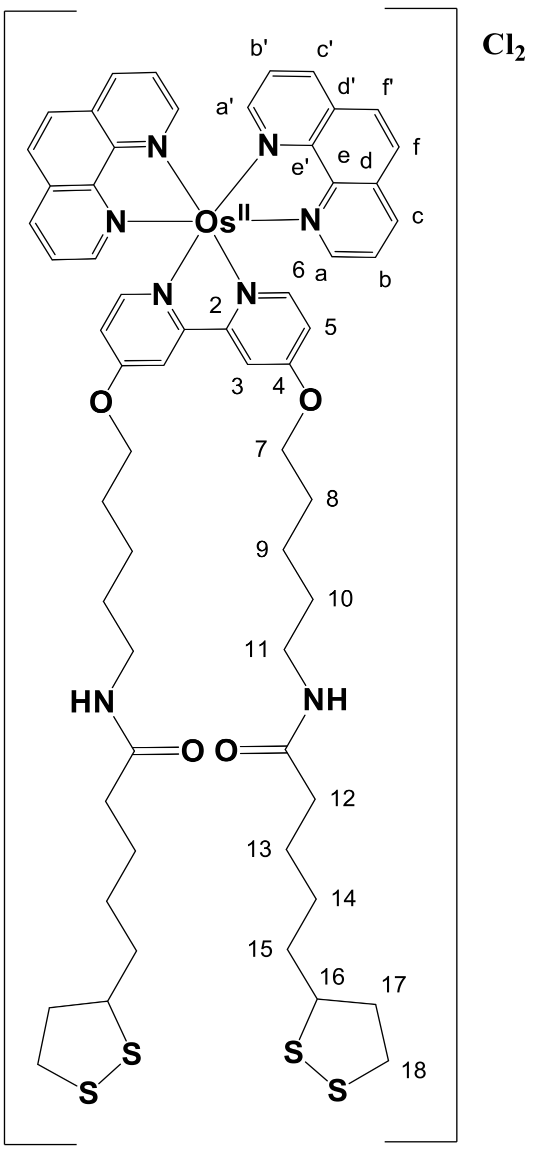

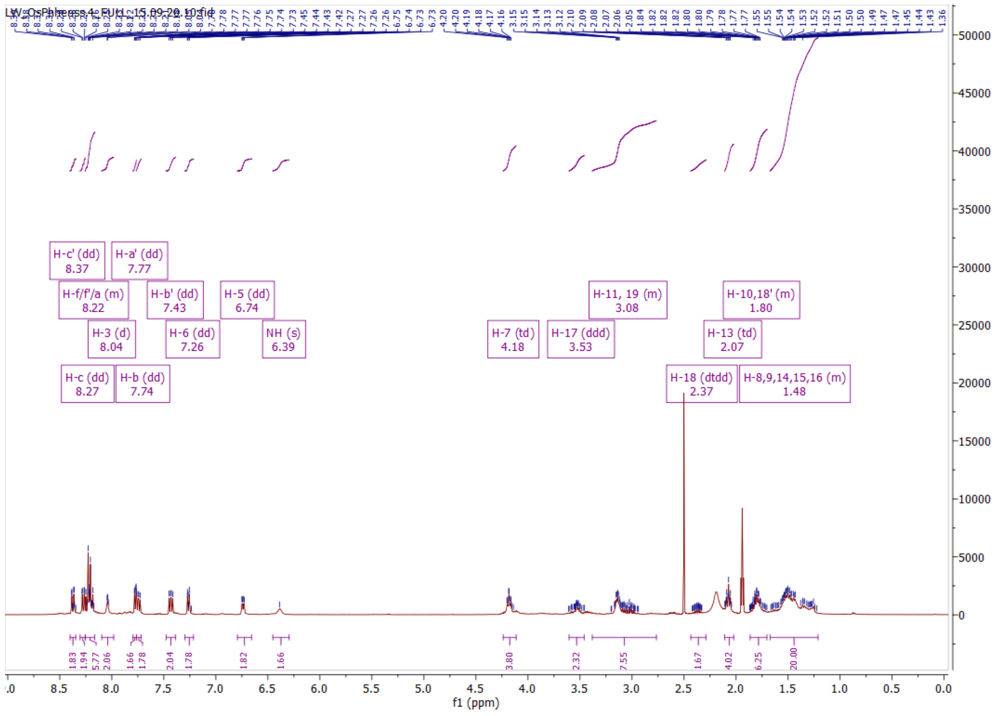

Figure S6. ^{1}H NMR spectrum of **OsPhenSS** in CD_3CN.

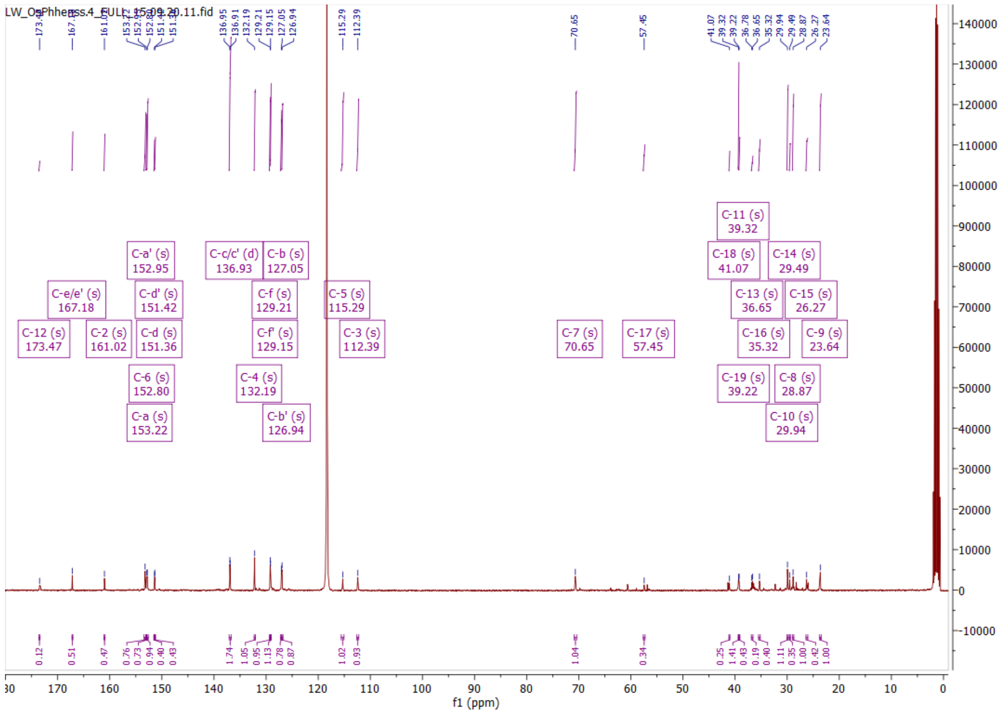

Figure S7. ^{13}C NMR spectrum of **OsPhenSS** in CD_3CN.

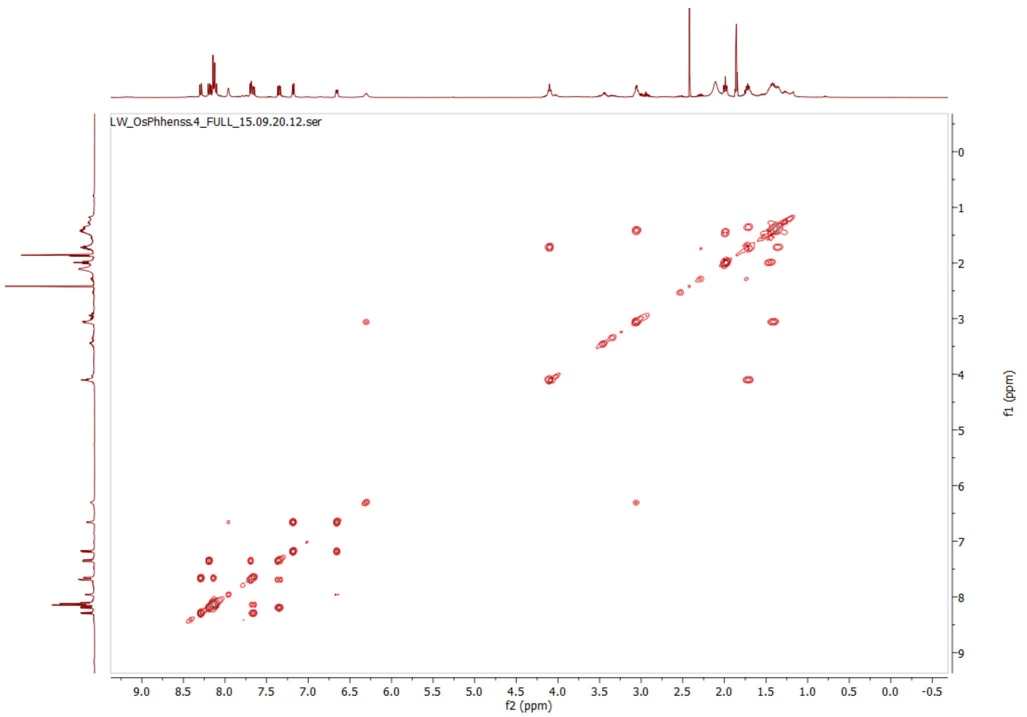

Figure S8. COSY spectrum of **OsPhenSS** in CD_3CN.

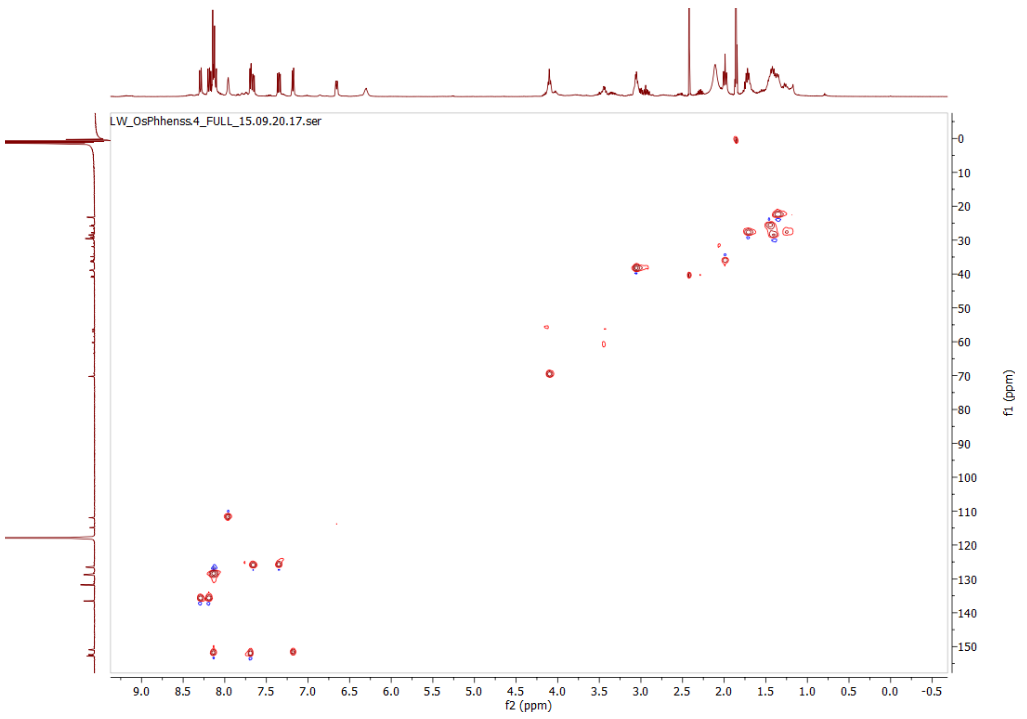

Figure S9. HSQC spectrum of **OsPhenSS** in CD_3CN.

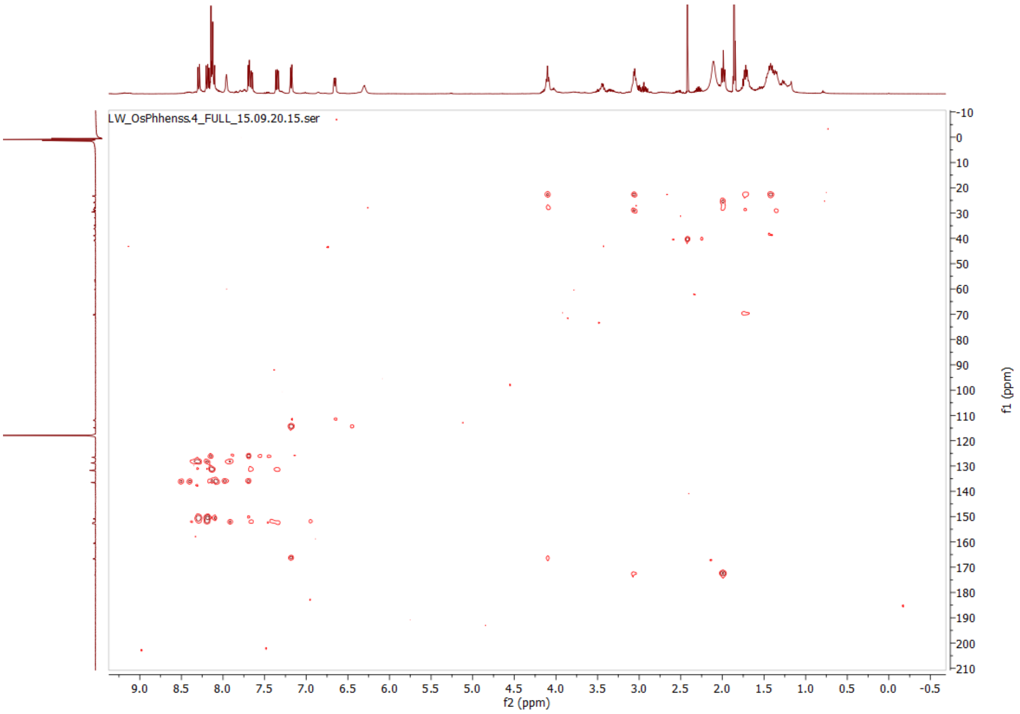

Figure S10. HMBC spectrum of **OsPhenSS** in CD_3CN.

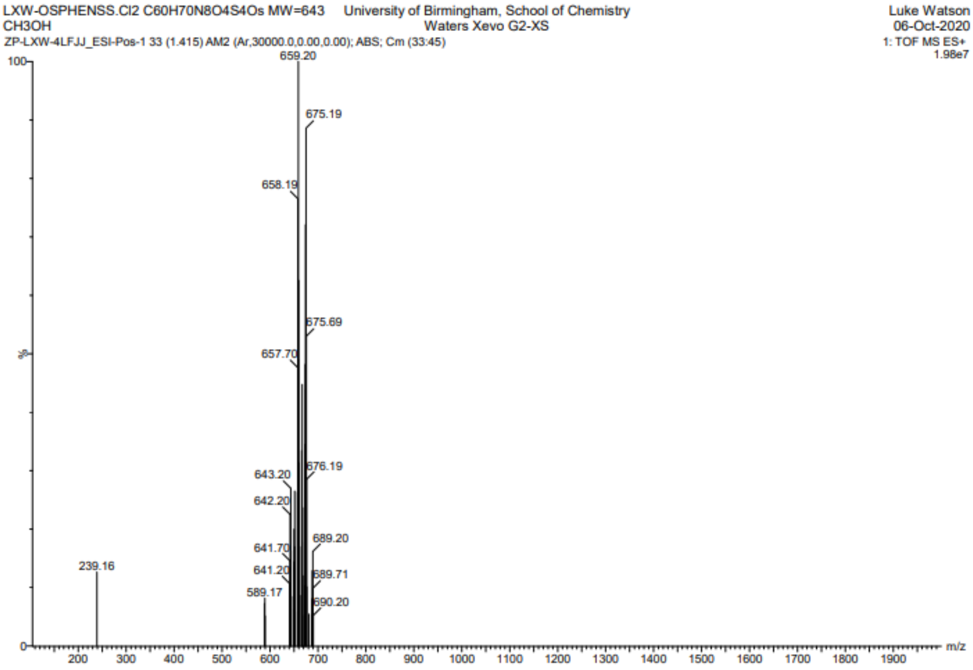

Figure S11. ESI (+) mass spectrum of **OsPhenSS**.

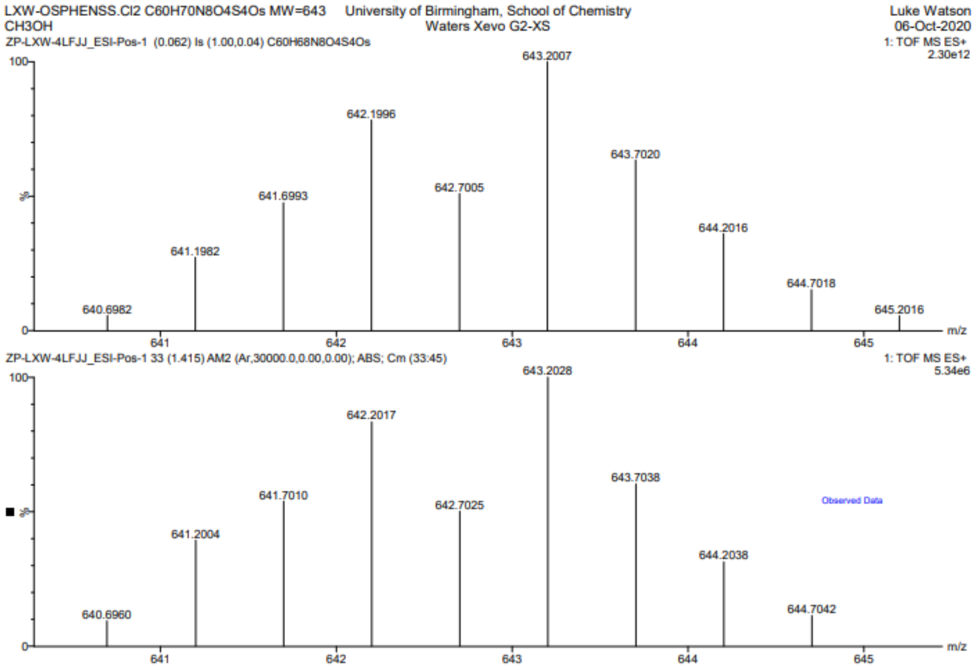

Figure S12. ESI (+) mass spectrum of **OsPhenSS.** Theoretical mass vs experimental exact mass.

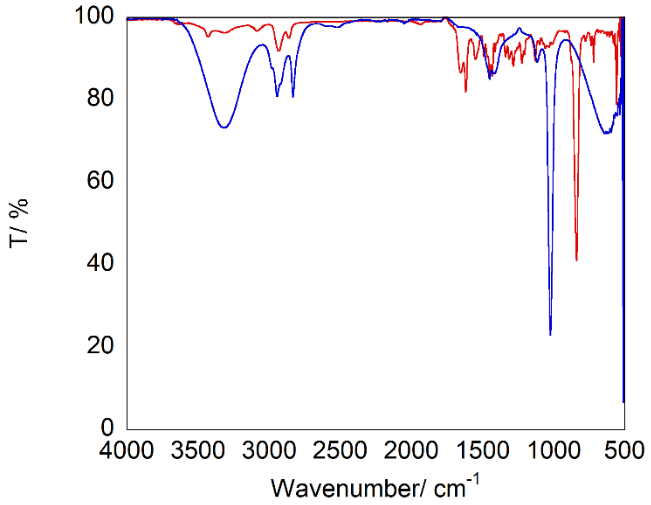

Figure S13. FTIR spectrum of **OsPhenSS** as the PF_6 counter ion (red) and the Cl counter ion (blue) showing successful ion exchange.

Photophysical Characterisation of OsPhenSS in presence of fluorosurfactant

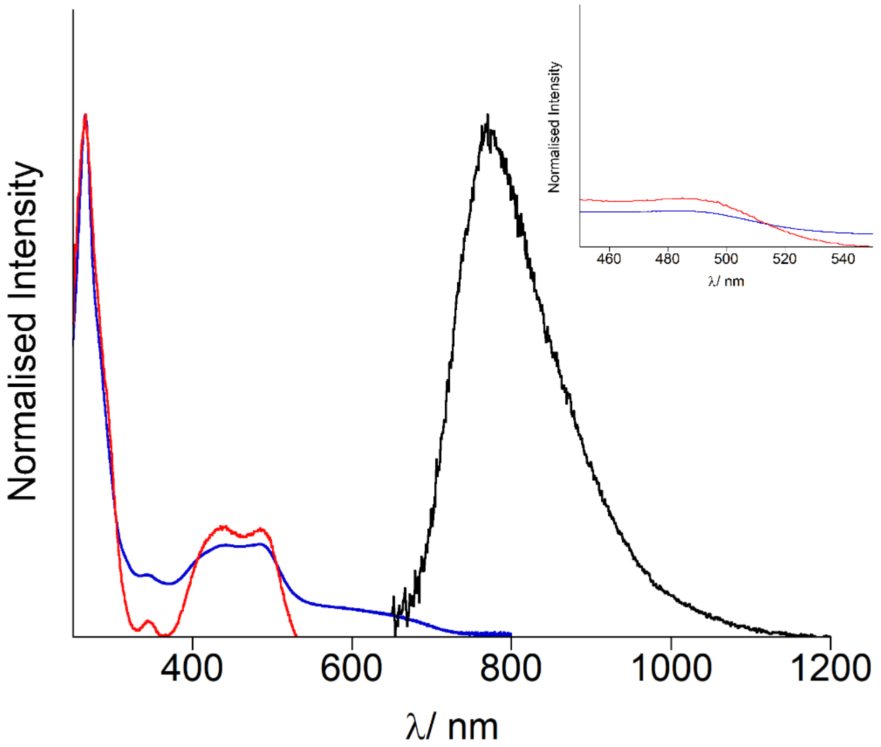

Figure S14. Normalised emission (black), excitation (red) and UV Vis (blue) of 10 µM **OsPhenSS** in the presence of 485 µM Zonyl FSA in 1mL aerated water (λ_{ex} = 485nm, λ_{em} = 770nm). Insert is magnification of excitation spectra 450 – 550 nm showing tail off.

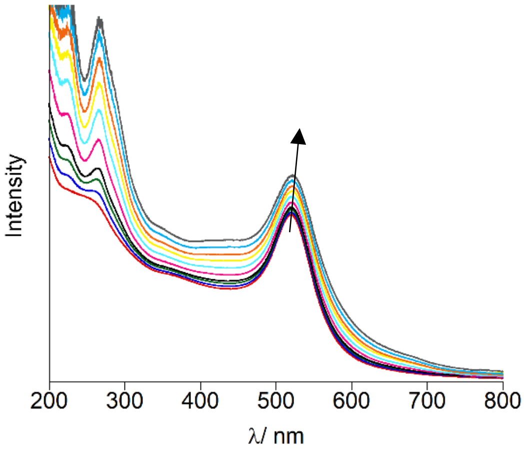

Figure S15. UV-Vis titration of 5 µL quantities of 1 mM **OsPhenSS** into 2 nM **Z•AuNP25** in water (35 µL added).

Data summary of gold nanoparticles' characterisation data

(A)

	λ_{max} / nm	Shift / nm
**AuNP13**	517	0
**Z•AuNP13**	518	1
**OsPhenSS•AuNP13**	521	4
**AuNP25**	518	0
**Z•AuNP25**	519	1
**OsPhenSS•AuNP25**	524	6

(B) **(C)**

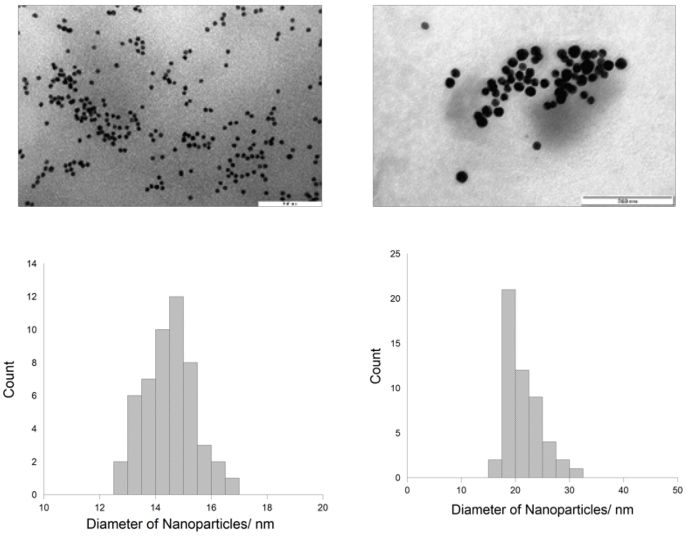

Figure S16. (A) Summary of UV-Vis shifts of gold nanoparticle samples; TEM images of (B) **OsPhenSS•AuNP13** and (C) **OsPhenSS•AuNP25** and associated histogram plots (n = 50). Scale bar = 200 nm.

(A)

	Number/ nm	Volume/ nm	PDI	Zeta potential / mV
**AuNP13**	14 ± 3	17 ± 4	0.06	-35 ± 4
**Z•AuNP13**	20 ± 5	23 ± 5	0.12	-49 ± 5
**OsPhen•AuNP13**	18 ± 5	31 ± 14	0.27	-39 ± 8
**AuNP25**	21 ± 5	25 ± 7	0.07	-34 ± 4
**Z•AuNP25**	29 ± 8	34 ± 13	0.26	-42 ± 4
**OsPhen•AuNP25**	27 ± 8	40 ± 25	0.32	-36 ± 3
**AuNP13 in D_2O**	13 ± 3	16 ± 6	0.095	-32 ± 8
**Z•AuNP13 in D_2O**	21 ± 6	27 ± 10	0.271	-76 ± 12
**OsPhenSS•AuNP13 in D_2O**	22 ± 6	28 ± 11	0.265	-36 ± 8

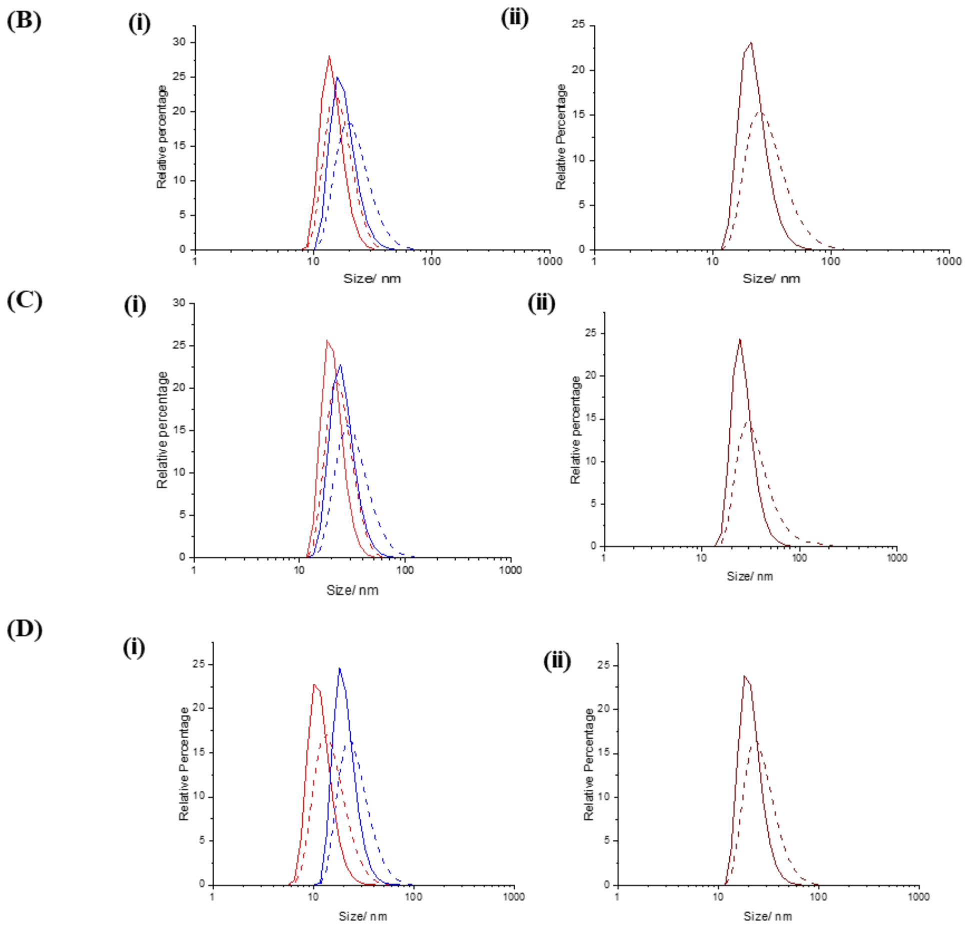

Figure S17. (A) Summary of Dynamic Light Scattering (DLS)data of gold nanoparticle samples (n = 5 for size and n = 3 for ζ-potential); **(B)** Number (solid line) an Volume (dashed line) size distributions for (i) citrate-capped **AuNP13** (red) and **Z•AuNP13** (blue) and (ii) **OsPhen•AuNP13** (brown); (C) Number (solid line) an Volume (dashed line) size distributions for (i) citrate-capped **AuNP25** (red) and **Z-AuNP25** (blue) and (ii) **OsPhen•AuNP25** (brown); (D) Number (solid line) an Volume (dashed line) size distributions for (i) citrate-capped **AuNP13** in D_2O **(**red) and **Z•AuNP13** in D_2O (blue) and (ii) **OsPhen•AuNP13** in D_2O (brown).

Photophysical characterisation of OsPhenSS•AuNP25

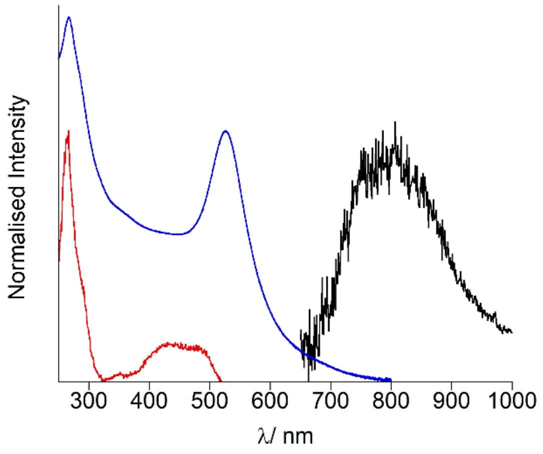

Figure S18. Normalised emission (black, λ_{exc} = 485 nm), excitation (red, λ_{em} = 785 nm) and UV-Vis (blue) spectra of **OsPhenSS•AuNP25** (2 nM) in aerated water.

Cell viability assays of OsPhenSS•AuNP in live A549 cells

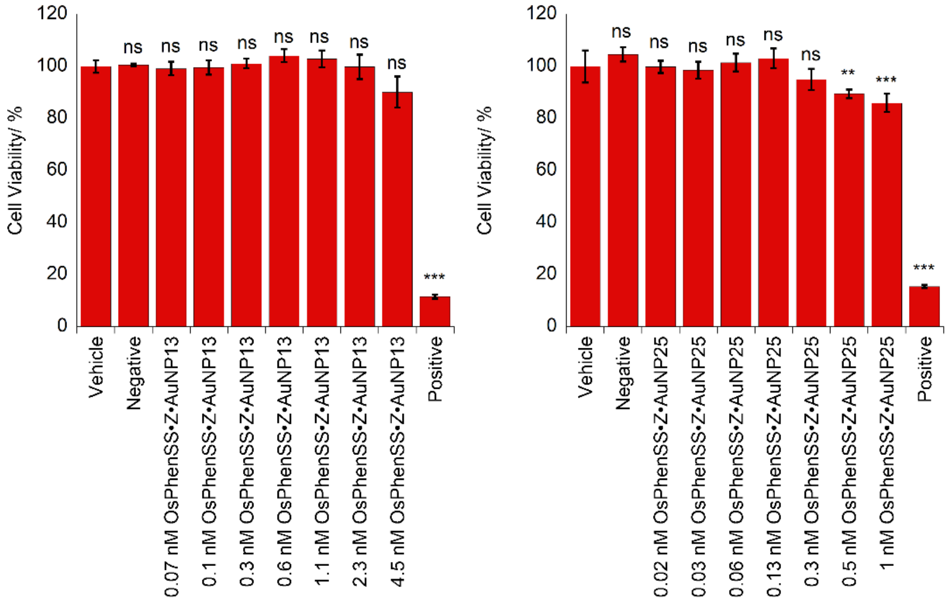

Figure S19. MTT assays of A549 cells treated with serial particle dilutions of (a) **OsPhenSS•AuNP13** and (b) **OsPhenSS•AuNP25** for 24 hours at 37°C 1-way ANOVA followed by a Dunnett's t-test corrected for multiple comparisons. *=P<0.05, **=P<0.01, ***=P<0.001, n = 3.

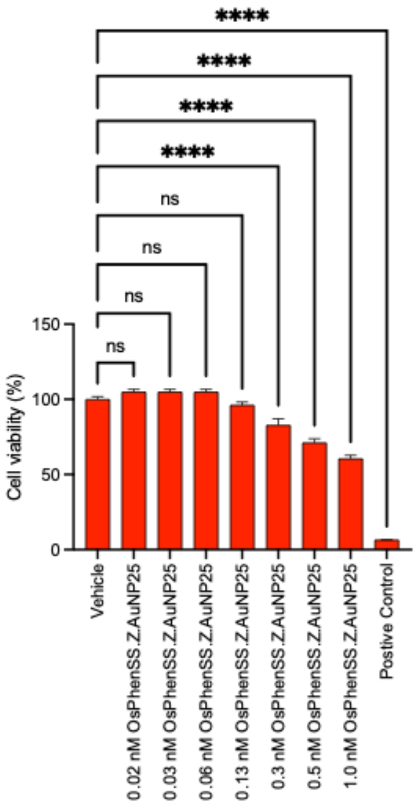

Figure S20. MTT cytotoxicity assay of MRC5 cells (embryonic human lung fibroblasts) treated with **OsPhenSS•AuNP25** for 24 h. Data is plotted as percentage control relative to vehicle alone. As a positive control cells were treated with 0.1% w/v Triton-X-100 for 24 h. The results represent the mean +/-SEM of six technical replicates. ****, statistically significant from vehicle control as assessed by a one-way ANOVA followed by a Dunnett's post-hoc t-test corrected for multiple comparisons, P<0.0001.

Additional Confocal microscopy images of OsPhenSS•AuNP25

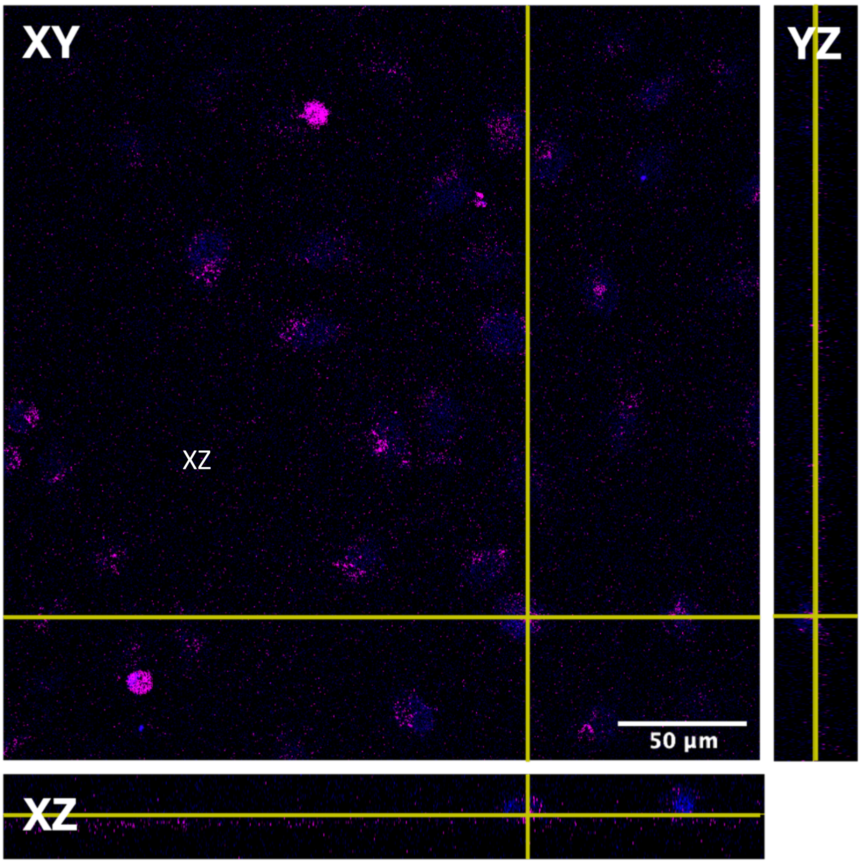

Figure S21. Orthogonal reconstruction of selected central plane from z-stack images of A549 cells showing osmium signal inside cells after incubation with 0.2 nM **OsPhenSS•AuNP25** for 18 h at 37°C. Purple channel, osmium emission (λ_{exc} = 488 nm, λ_{em} = 650 – 800 nm); blue channel, Hoescht 3368 nuclear staining (λ_{exc} = 402 nm, λ_{em} 420–470 nm).

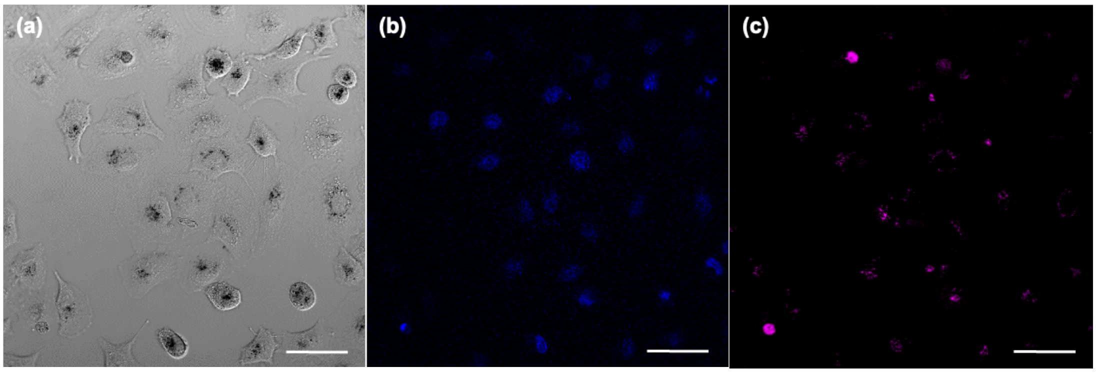

Figure S22. Separate channels (Figure 5) in the live cell images of A549 cells incubated with **OsPhenSS•AuNP25** (0.2 nM, incubation for 18 h). (a) brightfield, (b) blue channel, Hoescht 3368 nuclear staining (λ_{exc} = 405 nm, λ_{em}= 420 – 470 nm), (c) purple channel, osmium-based emission (λ_{exc} = 488 nm, λ_{em} = 650–800 nm). Scale bar is 50 µm.

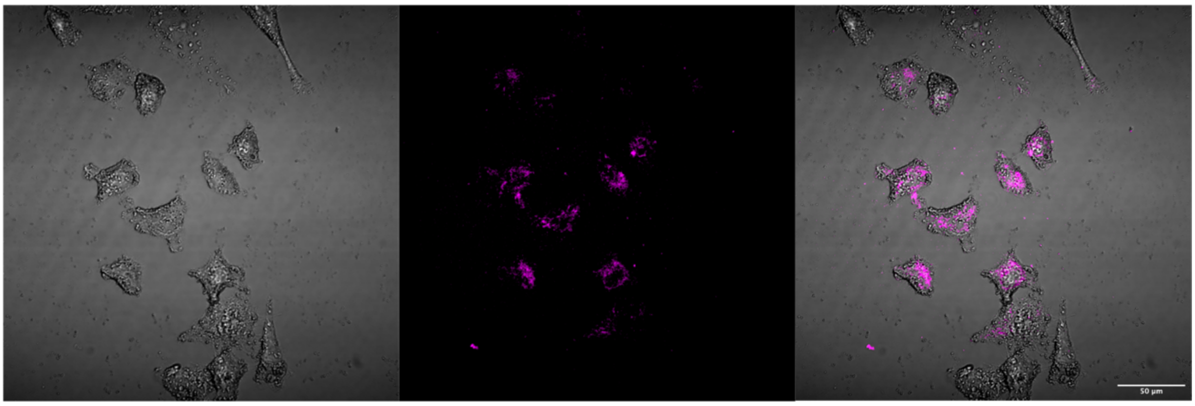

Figure S23. Confocal microscopy of **OsPhenSS•AuNP25** in fixed A549 cells (0.2 nM **OsPhenSS•AuNP25** at 4 h). Brightfield (left); Purple channel, **OsPhenSS•AuNP25** emission (λ_{exc} = 488 nm, λ_{em} = 650−800 nm) (middle) and overlap of brightfield and osmium emission (right). Scale bar is 50 µm.

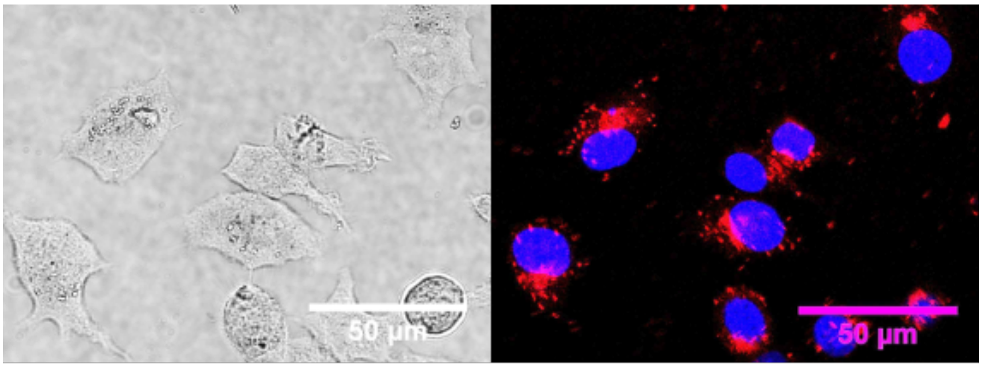

Figure S24. Dual channel fluorescence image of MCF-7 cells with corresponding brightfield image incubated with **OsPhenSS•AuNP25** at 0.1 nM incubated for 24 h (red channel, λ_{exc} = 552 nm, λ_{em} = 680-710 nm) and Nuclear Violet (Cambridge Bioscience, UK) incubated for 1 h at 10µM prior to imaging (blue channel, λ_{exc} = 405 nm, λ_{em} = 430-470 nm) to stain the nucleus. The luminescence shows punctate intracellular and perinuclear distribution. Scale bar is 50 µm.

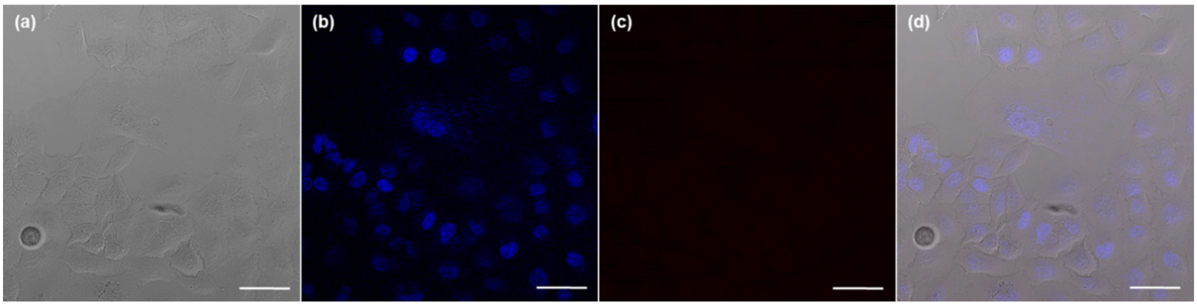

Figure S25. Confocal image of untreated control A549 cells. (a) Brightfield, (b) blue channel, Hoechst 3368 nuclear staining (λ_{exc} = 405 nm, λ_{em}= 420 − 470 nm), (c) purple channel, osmium (λ_{exc} = 488 nm, λ_{em} = 650 − 800 nm) and (d) overlap of brightfield, blue channel (Hoechst 3368) and purple channel (osmium). Scale bar is 50 µm.

Calculation of number (*N*) of gold atoms per nanoparticle

Assuming the gold nanoparticles are perfect spheres and atoms are packed in a uniform face-centred cubic crystalline structure, and taking the density of gold as $\rho_{gold} = 19.32$ g cm^{-3}

$$\boldsymbol{N} = \frac{\boldsymbol{\rho \text{ x } \pi D^3}}{\boldsymbol{6 \text{ x } M_r}} \text{ x } N_A$$

Where Mr of gold =196.96657 g mol^{-1}, Avogadro's constant $N_A = 6.022140857 \times 10^{23}$ mol^{-1}

The equation simplifies:

$$N = 30.8969 \times D^3$$
